# Fengycin–essential oil emulsions as sustainable biocontrol formulations against *Moniliophthora roreri*, the cacao frosty pod rot pathogen

**DOI:** 10.3389/fpls.2025.1731535

**Published:** 2026-01-16

**Authors:** Mirian Villavicencio-Vásquez, Fernando Espinoza-Lozano, Ivan Chóez-Guaranda, Valeria Cos-Farias, Jonathan Coronel-León

**Affiliations:** 1 Centro de Investigaciones Biotecnológicas del Ecuador (CIBE), Escuela Superior Politécnica del Litoral (ESPOL), Guayaquil, Ecuador; 2 Facultad de Ciencias Naturales y Matemáticas, Escuela Superior Politécnica del Litoral (ESPOL), Guayaquil, Ecuador; 3 Facultad de Ingeniería Mecánica y Ciencias de la Producción (FIMCP), Escuela Superior Politécnica del Litoral (ESPOL), Guayaquil, Ecuador

**Keywords:** biological control, emulsions, essential oils, fengycin, *Moniliophthora roreri*

## Abstract

**Introduction:**

Moniliophthora roreri, the causal agent of cacao frosty pod rot (FPR), represents a major constraint to cacao production in tropical regions. The increasing demand for eco-compatible crop protection strategies highlights the need for synergistic bioformulations combining microbial and plant-derived antifungal agents.

**Methods:**

In this study, we evaluated the antifungal activity and physicochemical stability of emulsions formulated with fengycin, a cyclic lipopeptide produced by *Bacillus subtilis* DS03, and essential oils (EOs) from cinnamon (*Cinnamomum zeylanicum*) and peppermint (*Mentha piperita*). Fengycin isoforms were identified by mass spectrometry (m/z 1435–1491). Antifungal activity against *M. roreri* was assessed individually and in combination using inhibition assays, fractional inhibitory concentration (FIC) indices, and colloidal stability measurements (ζ-potential).

**Results:**

Fengycin inhibited mycelial growth of *M. roreri* by 84.6% at 1000 ppm, while cinnamon EO achieved complete inhibition at concentrations ≥500 ppm. Fengycin–cinnamon (F–C) emulsions showed strong synergistic antifungal activity (FIC < 0.5) and high colloidal stability (ζ = –26 to –35 mV). In contrast, fengycin–peppermint (F–P) emulsions exhibited additive effects (FIC ≈ 1).

**Discussion:**

The enhanced antifungal efficacy of F–C emulsions is attributed to the dual role of fengycin as both an antifungal compound and a natural biosurfactant, which improves the dispersion and bioavailability of cinnamaldehyde-rich essential oils. These findings demonstrate the potential of a stable, bio-based antifungal formulation that integrates microbial and plant metabolites, offering a scalable and sustainable strategy for controlling fungal diseases in cacao and other crops.

## 1 Introduction

Cacao (*Theobroma cacao* L.) is the primary raw material for chocolate production, a globally expanding industry. This crop plays a crucial role in several countries’ agricultural and economic development, including Ecuador, Côte d’Ivoire, Ghana, Indonesia, Nigeria, Mexico, and Brazil (Kongor et al., 2024). Cacao cultivation in Ecuador contributes significantly to rural livelihoods and national income (Dahunsi et al., 2019; Chamba et al., 2024). However, cacao productivity is severely threatened by Frosty Pod Rot (FPR), caused by *Moniliophthora roreri*, which can result in crop losses of up to 90% in affected regions of the Americas (Bailey et al., 2018) and approximately 60% in Ecuador (Jiménez et al., 2022). Conventional control strategies, such as pruning, drainage, and removing infected pods, are only partially effective. Moreover, fungicide applications have led to environmental contamination, resistant strains, and increased production costs (Yin et al., 2023). Given these limitations, there is growing interest in sustainable alternatives, including biocontrol agents, disease-resistant cacao clones, microbial biosurfactants, and plant-derived products such as essential oils (EOs) (Silva et al., 2024; Villavicencio-Vásquez et al., 2025). EOs are complex mixtures of volatile compounds with antimicrobial properties against numerous phytopathogens (Shaaban, 2020). Among these, peppermint (*Mentha piperita*) and cinnamon (*Cinnamomum zeylanicum*) EOs are consistently reported as effective antifungal agents (Martins and Bicas, 2024). Their efficacy is attributed to bioactive constituents such as menthol, menthone, linalool, cinnamaldehyde, and eugenol (Mohammed et al., 2022; Yu, 2025). Despite their potential, EO application in agriculture is limited by low water solubility, volatility, and phytotoxicity (Chang et al., 2022). Emulsification improves stability and delivery, but the choice of surfactant is critical for maintaining biological activity (Guillamon et al., 2024).

Among available surfactants are microbial surfactants or biosurfactants, particularly lipopeptides produced by *Bacillus* spp. stand out for their biodegradability, surface activity, and antifungal properties (Valenzuela-Ruíz et al., 2020). Within this group, fengycin, a cyclic lipopeptide from *Bacillus subtilis*, has been recognized for its strong antifungal activity and ability to induce systemic resistance in plants (Farzand et al., 2019; Yánez-Mendizábal and Falconí, 2021). Previous studies have shown that *B. subtilis* strains can inhibit *M. roreri* growth by up to 95% (Guato-Molina et al., 2019). Nevertheless, the structural confirmation of fengycin and potential synergistic interactions with EOs against *M. roreri* remain poorly explored.

Based on previous evidence, the strain *B. subtilis* DS03 has been identified as a potent producer of microbial surfactants with strong emulsifying capacity in oil-in-water systems and notable surface tension reduction (Mendoza et al., 2022). In this context, our research team hypothesizes that emulsions combining fengycin with cinnamon EO could exhibit superior antifungal efficacy and formulation stability compared to individual applications. Therefore, this study aims to confirm the presence of fengycin in *B. subtilis* DS03 using mass spectrometry and to evaluate its synergistic activity with cinnamon EO against multiple Ecuadorian isolates of *M. roreri*. As a result, this work provides mechanistic insights and practical implications for developing sustainable biocontrol strategies in cacao production through integrating chemical characterization, formulation analysis, and antifungal assays.

## 2 Materials and methods

### 2.1 Origin of *M. roreri* isolates

Eight isolates of *M. roreri* were selected from the preserved microbial collection of the Ecuadorian Center for Biotechnology Research (World Data Centre for Microorganisms, WDCM1151), based on their geographic origin from diverse cacao producing regions in Ecuador: MR24 and MR26 (Morona Santiago), MR34 (Zamora Chinchipe), MR50 (Sucumbíos), MR69 (Orellana), MR74 (Napo), MR82 (Pastaza), and MR95 (El Oro). The strains were reactivated on potato dextrose agar (PDA, BD Difco™) at 25 ± 1°C for 10 days before use in antifungal assays.

These isolates had been previously characterized for their differential sensitivity to the chemical fungicides Flutolanil and Azoxystrobin (Amaya-Márquez et al., 2021; Espinoza-Lozano et al., 2022). This geographic and phenotypic diversity is particularly relevant, as *M. roreri* populations exhibit considerable genetic and physiological variability depending on their region of origin. Such differences can influence key pathogenic traits, including virulence, resistance to chemical treatments, and responsiveness to biocontrol strategies. Incorporating this variability into the present study enables a more robust and realistic evaluation of the antifungal efficacy of the tested formulations under field-relevant conditions.

### 2.2 Production and characterization of lipopeptides from *B. subtilis* DS03

The strain *B. subtilis* DS03 was cultured in a mineral medium (MM) with the following composition (g/L): glucose 10, NaNO_3_ 8.5, CaCl_2_ 7×10^-6^, KH_2_PO_4_ 4, MgSO_4_.7H_2_O 0.21, Na_2_HPO_4_ 5.7, FeSO_4_.7H_2_O 0.01, and yeast extract 1. Cultures were incubated at 35°C for 48 hours with constant agitation at 110 rpm. Lipopeptides were recovered following the methodology described by Mendoza et al., 2022. The crude biosurfactant extracted from the culture medium was purified by column chromatography using silica gel (60 mesh; Merck, Darmstadt, Germany) and eluted with a solvent mixture of chloroform: methanol: water (65:25:4, v/v/v). Eluted fractions (1 mL each) were analyzed by thin-layer chromatography (TLC) on silica gel 60 G plates (Macherey-Nagel, Düren, Germany) using the same mobile phase. Fractions were visualized with ninhydrin reagent (Sigma, St. Louis, MO), which selectively detects amino groups, to confirm the presence of lipopeptides. Fractions with similar polarity and surface tension reducing activity were subjected to structural characterization by electrospray ionization tandem mass spectrometry (ESI-MS/MS) in positive-ion mode (LC/MSD-TOF; Agilent Technologies, CA, USA). Analyses were conducted with a capillary voltage of 3.5 kV, using nitrogen as both the nebulizing and drying gas, to determine the molecular weights of the lipopeptide components (Coronel-León et al., 2015).

### 2.3 GC-MS analysis of essential oils

The chemical composition of the EOs was determined using gas chromatography–mass spectrometry (GC-MS) on an Agilent Technologies system (7890A GC coupled to a 5975C inert XL MSD with a triple-axis detector). Separation was achieved using an HP-5 capillary column (30 m × 0.32 mm i.d., 0.25 µm film thickness; phenyl methyl siloxane as the stationary phase), with ultra-high purity helium as the carrier gas at a flow rate of 2.5 mL/min. Samples were prepared by dissolving the oils in diethyl ether (1:1), and 1.0 µL of each solution was injected in split mode (100:1) at 250°C. The oven temperature program began at 40°C, increasing at 3°C/min to a final temperature of 240°C. The transfer line temperature was maintained at 280°C, while the ion source operated at 230°C under electron ionization (EI) at 70 eV. Data were acquired in full scan mode (40–550 amu), with no solvent delay applied. Compound identification was performed by comparing the obtained mass spectra with those in the NIST 11 and Wiley 9 spectral libraries. Additionally, retention indices were calculated using a homologous series of n-alkanes (C7–C30) and compared with values reported in the NIST Chemistry WebBook. Standard saturated alkanes were obtained from Supelco, and diethyl ether from Fisher Scientific (Hampton, USA).

### 2.4 Antifungal activity bioassays of fengycin and EOs

The antifungal activity of fengycin and EOs was evaluated using the poisoned food technique. For fengycin, PDA was amended with the lipopeptide at 100, 250, 500, and 1000 ppm concentrations. Five-millimeter mycelial disks of *M. roreri* (obtained from 10-day-old cultures) were placed at the center of each plate. Plates were incubated at 25 ± 1°C for 10 days. Each treatment was performed with five replicates, and plates without fengycin were negative controls. The antifungal effect of fengycin was expressed as a percentage of mycelial growth inhibition (PGI), which was calculated using the following Equation 1 (Ezziyyani et al., 2004).

(1) $$\text{PGI}\left(\%\right)=\left[\left({\text{R}}_{1}–{\text{R}}_{2}\right)/{\text{R}}_{1}\right]\times 100$$

where R_1_ is the radial growth (mm) of the fungus in the control, and R_2_ is the radial growth in the presence of fengycin.

The EOs commercially available cinnamon and peppermint EOs (NOW^®^ Foods, USA) were prepared as a 165.000 ppm stock solution in dimethyl sulfoxide (DMSO). From this stock, an intermediate dilution of 2000 ppm was prepared using sterile distilled water, which was then used to obtain final concentrations of 100, 250, 500, 1000, and 2000 ppm. The final DMSO concentration in the treatments did not exceed 1% (v/v). EO solutions at each concentration were incorporated into PDA cooled to 45 ± 1°C, poured into sterile Petri dishes, and allowed to solidify. Five-millimeter mycelial disks of *M. roreri* were placed at the center of each plate. Plates were incubated at 25 ± 1°C for 10 days, with five replicates per treatment. Sterile distilled water was used as a negative control. The antifungal activity of the EOs was expressed as PGI (%) and calculated using the same formula described above.

### 2.5 Antifungal activity of the emulsions fengycin and EOs

To investigate the potential antifungal effects of combined formulations, emulsions of fengycin and EOs were prepared using the spontaneous emulsification method, in which emulsification occurs through the controlled mixing of immiscible organic and aqueous phases under specific physicochemical conditions (Mohammed et al., 2022). The concentrations selected corresponded to the minimum inhibitory concentrations (MICs) previously identified as effective against *M. roreri.* For each concentration, five fixed-ratio mixtures (C1–C5) were formulated to evaluate interaction effects between fengycin and the EOs. These ratios were defined as 0/100, 25/75, 50/50, 75/25, and 100/0 (fengycin%/EO%) allowing evaluation of potential synergistic or additive effects. These emulsions were then tested for antifungal activity following the radial mycelial growth inhibition assay described previously, and their effect on spore germination. All experiments were performed with five replicates.

To determine potential synergistic interactions between fengycin and EOs, the Fractional Inhibitory Concentration (FIC) index was calculated using the following Equation 2 described by (Coronel-León et al., 2017).

(2) $$\text{FIC}=\left[\left({\text{MIC}}_{EOM}/{\text{MIC}}_{EO}\right)+\left({\text{MIC}}_{FM}/{\text{MIC}}_{F}\right)\right]$$

Where MIC_EOM_ is the MIC of the EO in the emulsion mixture, MIC_EO_ is the MIC of the EO alone, MIC_FM_ is the MIC of fengycin in the emulsion mixture, and MIC_F_ is the MIC of fengycin when used alone. An FIC ≤ 0.5 indicated synergism. In contrast, values from 0.5 to 1 indicated no synergism, between 1 and 4 were defined as indifferent, and higher than four indicated antagonism (Bidaud et al., 2021).

### 2.6 Characterization and stability of antimicrobial emulsions

The physical properties of the fengycin-peppermint (F-P) and fengycin-cinnamon (F-C) emulsions that demonstrated the highest antifungal activity (at 1000 ppm and 250 ppm, respectively) were evaluated using dynamic light scattering (DLS) with a Zetasizer Nano (Malvern Instruments). This analysis included measurements of droplet size (Z-average diameter, Dz) and zeta potential (ζ), which reflect the electrokinetic stability of the formulations. All measurements were performed at 25°C. These parameters are critical for evaluating emulsion quality and predicting long-term physical stability. To assess the temporal stability of the formulations, DLS measurements were recorded weekly over a seven-week period (weeks 1 to 7) under controlled storage conditions (temperature: 25 ± 1°C; relative humidity: 50 ± 5%). Any significant variations in size and ζ-potential were tracked to determine potential instability and to support formulation optimization for future antifungal applications.

### 2.7 Spore germination inhibition assay

The inhibitory activity of fengycin and EOs on spore germination of *M. roreri* was evaluated using a spore suspension adjust to 2×10^5^ spores/mL (Chen et al., 2017; Villegas-Rascón et al., 2018; Porcino et al., 2023; Lam-Gutiérrez et al., 2024; Sanchez-Tamayo et al., 2024) prepared from 10-day-old cultures grown on PDA at 25 ± 1°C. A volume of 50 µl of the suspension was plated onto PDA medium amended with the following treatments: pure fengycin (100, 250, and 1000 ppm), cinnamon EO (100 and 250 ppm), peppermint EO (1000 ppm), binary mixtures of F–C (100 and 250 ppm), and an F-P mixture (1000 ppm). Plates were incubated at 25 ± 1°C for 5 days, after which spore germination (defined as the initiation of mycelial growth from spores) was quantified. For each treatment, 50 spores were evaluated per plate, with three replicates per treatment. Spore concentration was determined using a Neubauer hematocytometer under a light microscope at 40x magnification, and the percentage of spore germination inhibition (SGI) was calculated using the Equation 3 described by Ribes et al. (2017).

(3) SGI(%)=(sc–st)/sc×100

Where sc is the average number of germinated spores in the control, and st is the number in the treated condition.

### 2.8 Statistical analysis

Mycelial growth inhibition data were analyzed using a one-way ANOVA, considering each treatment concentration separately and comparing the responses of the different *M. roreri* isolates. Significant differences between means were determined using Tukey’s multiple comparison test (p< 0.05). This approach allowed for identifying of variations in strain sensitivity to the different concentrations tested. Different lowercase letters indicate statistically distinct groups.

To assess the combined effect of fengycin and EOs, one-way ANOVA and Tukey’s HSD test (p< 0.05) were also applied, and results are presented as grouped bar plots. Boxplots and interaction plots were also generated to illustrate central tendency, variability, and the relationship between treatment type and concentration on fungal inhibition across multiple *M. roreri* strains.

**Figure 1:**
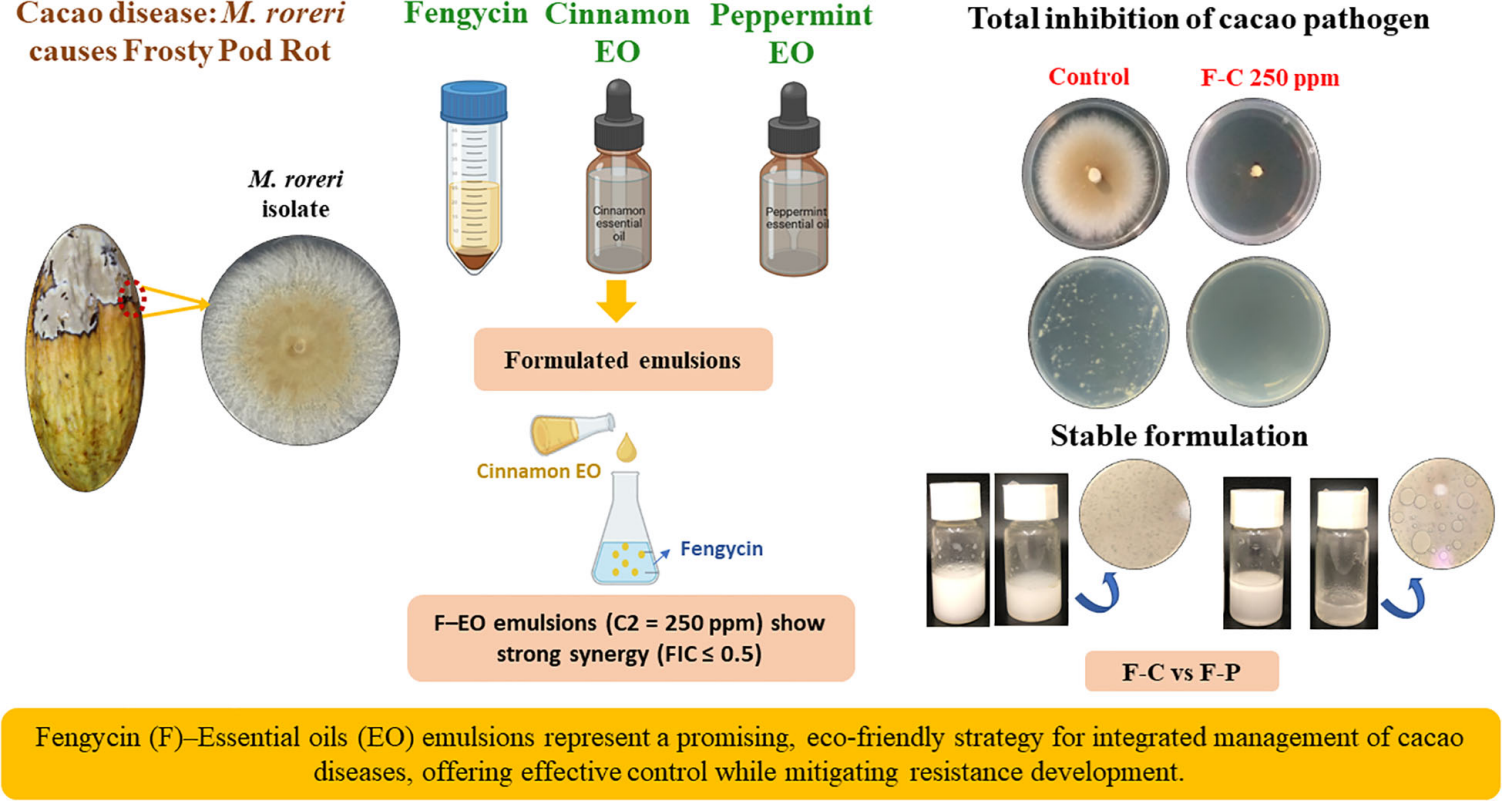
Schematic overview of the workflow for antifungal evaluation and formulation development. Illustration summarizing the experimental process: (i) collection of infected cacao pods and isolation of M. roreri on PDA plates; (ii) preparation of pure fengycin, essential oils (EOs), and mixed fengycin–EO emulsions; (iii) *in vitro* assays to assess mycelial growth and spore-germination inhibition; and (iv) selection of the most effective and physically stable formulations. This schematic integrates the methodological sequence and the main antifungal outcomes obtained in the study. Created using Biorender.

A schematic overview summarizing the entire methodological workflow is presented in **Figure 1**, providing a visual outline of the process from fungal isolation to emulsion preparation and antifungal evaluation. All analyses were performed using InfoStat v2024 (Grupo InfoStat, FCA, Universidad Nacional de Córdoba, Argentina). Data are reported as mean ± standard deviation (SD), and statistical significance was established at p< 0.05.

## 3 Results

### 3.1 Fengycin production

**Figure 2:**
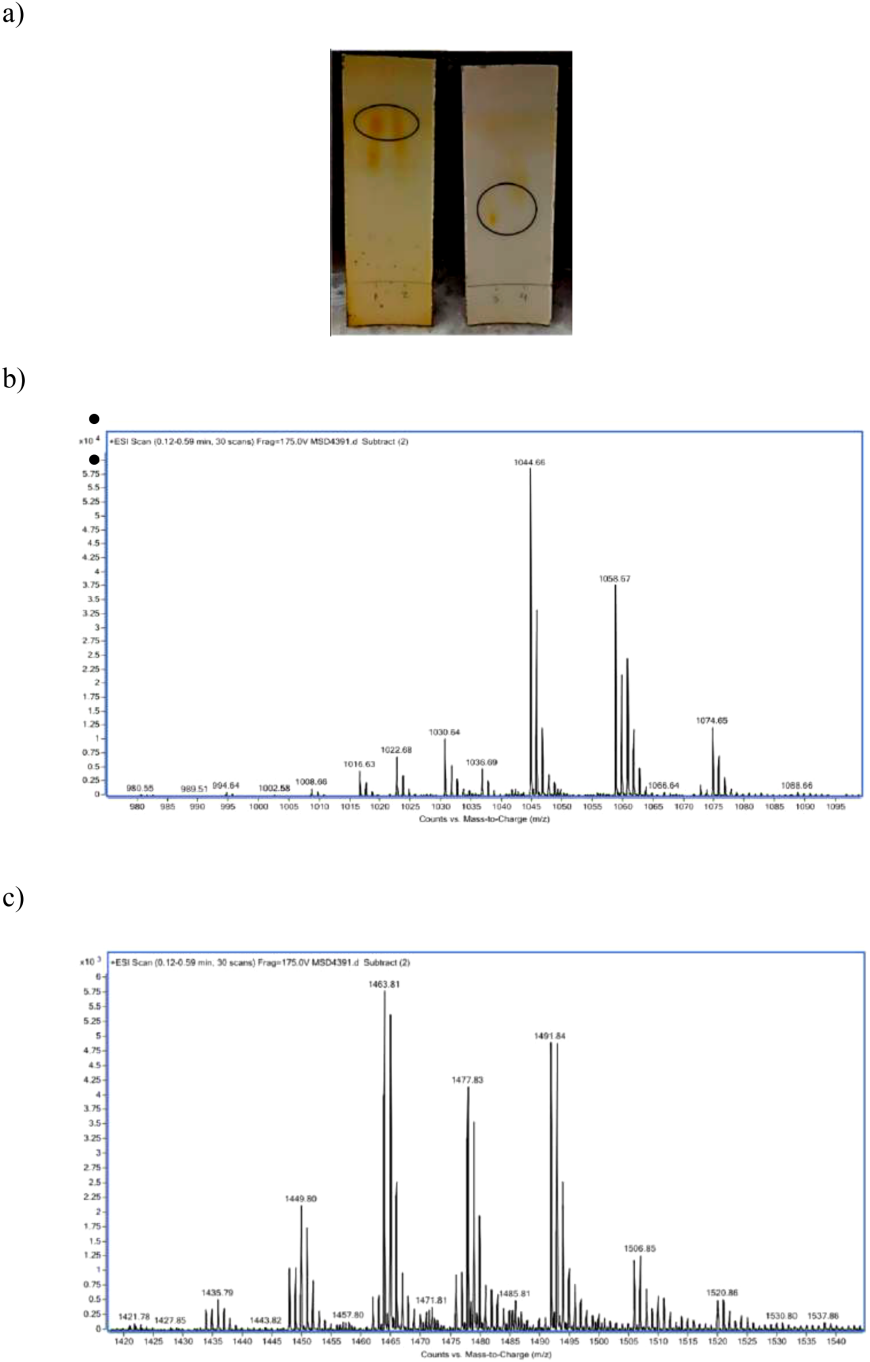
Separation and analysis of fengycin-containing fractions by thin-layer chromatography (TLC). TLC profile of the crude biosurfactant extract showing separation of components using silica gel and a chloroform: methanol: water (65:25:4, v/v/v) mobile phase. Visualization was performed with ninhydrin reagent to detect amino group–containing lipopeptides **(A)**. Fraction A (1-2): early eluting compound with limited surface activity **(B)**. Fraction B (3-4): major lipopeptide-enriched fraction, identified as fengycin based on subsequent antifungal activity and ESI-MS/MS analysis **(C)**.

The biosurfactant produced by *B. subtilis* DS03 was recovered by acid precipitation and initially characterized by TLC, showing distinct ninhydrin-positive spots (**Figure 2A**) consistent with a lipopeptide nature. Silica gel column yielded 45 fractions, grouped into two main categories: Fraction A (fractions 1–2), characterized by higher hydrophobicity, while Fraction B (fractions 3–4) showed greater polarity. Both fractions significantly reduced surface tension, from 67 mN/m to 32 mN/m and 30 mN/m.

Mass spectrometry confirmed the identity of Fraction A as fengycin, with [M+H]+ ions at m/z 1435, 1449, 1463, 1477, and 1491, and sodium adducts at m/z 1457, 1471, and 1485 (**Figure 2B**). These values correspond to fengycin isoforms A and B (C14–C16 fatty acid chains), consistent with reported molecular weights (1400–1500 Da). Fraction B was identified as surfactin, with [M+H]+ ions at m/z 1008, 1022, and 1036, and [M+Na]+ adducts at m/z 1030, 1044, and 1058 (**Figure 2C**).

Fraction A (fengycin) was selected for subsequent antifungal assays and emulsion formulations.

### 3.2 Essential oils composition

A total of 40 volatile organic compounds were identified in cinnamon EO, representing 98.09% of the analyzed sample. These included one alcohol, six aldehydes, one benzodioxole, two esters, one ketone, 12 monoterpenes, six oxygenated monoterpenes, eight sesquiterpenes, one oxygenated sesquiterpene, one phenylpropene, and one styrene. The predominant constituents were (E)-cinnamaldehyde (69.72 ± 0.15%) and eugenol (4.02 ± 0.02%).

In the case of peppermint EO, 35 volatile compounds were detected, accounting for 97.88% of the sample. These included two alcohols, one ketone, seven monoterpenes, 14 oxygenated monoterpenes, 10 sesquiterpenes, and one oxygenated sesquiterpene. The major components were menthol (51.07 ± 0.01%), menthone (17.12 ± 0.01%), neoisomenthol (10.58 ± 0.01%), eucalyptol (6.06 ± 0.01%), and menthyl acetate (4.18 ± 0.01%).

Cinnamon EO was rich in aldehydes (71.1%), whereas peppermint oil was characterized by oxygenated monoterpenes (95.2%) (**Supplementary Tables S1, S2**). These dominant constituents are known for strong antifungal activity, suggesting differential inhibition mechanisms.

### 3.3 Antifungal activity of fengycin and EOs

The EOs of cinnamon and peppermint, along with the microbial surfactant fengycin, demonstrated inhibitory effects against the mycelial growth of eight *M. roreri* strains, with efficacy depending on concentration (**Table 1**). Statistically significant differences were observed between treatments (p< 0.05).

**Table 1:** Antifungal activity of peppermint, cinnamon, and fengycin against *M. roreri*.

Concentrations (ppm)	Peppermint (%)	Cinnamon (%)	Fengycin (%)
100	9.05 ± 3.39 a	5.24 ± 3.13 a	43.60 ± 7.68 a
250	12.02 ± 4.77 a	84.89 ± 22.84 b	63.25 ± 4.52 b
500	23.44 ± 7.00 b	100 ± 0.00 c	75.04 ± 3.48 c
1000	77.99 ± 5.52 c	100 ± 0.00 c	84.57± 3.28 d
2000	100 ± 0.00 d	100 ± 0.00 c	——–

(Mean values ± SD, different letters indicate significant differences at p< 0.05, Tukey test).

Peppermint EO showed a dose-dependent antifungal response, increasing from 9.05% inhibition at 100 ppm to 100% at 2000 ppm. Its antifungal properties are associated with menthol (51.07%) and menthone (17.12%), which exert their effects by disrupting fungal plasma membranes, leading to leakage of intracellular contents and cell lysis. Although less potent than cinnamon EO at low concentrations, peppermint EO proved highly effective at higher doses.

Fengycin, a cyclic lipopeptide produced by *B. subtilis* DS03, also demonstrated consistent inhibitory activity across all concentrations tested, with a maximum inhibition of 84.57% at 1000 ppm. Fengycin targets fungal pathogens by integrating into the lipid bilayer of the fungal membrane, where it forms pores or destabilizes the structure, leading to membrane disruption and cell death. Its selectivity for sterol-rich membranes makes it particularly effective against filamentous fungi like *M. roreri.*

These findings suggest that the antifungal efficacy of each treatment is closely related to the chemical nature and dominant bioactive compounds of the EOs or microbial surfactant. While cinnamon oil was effective at low concentrations due to the strong activity of cinnamaldehyde, peppermint required higher doses for complete inhibition. Fengycin showed robust and broad-spectrum activity, making it a promising alternative or complement to plant-based products in the biological control of *M. roreri.*

**Figure 3:**
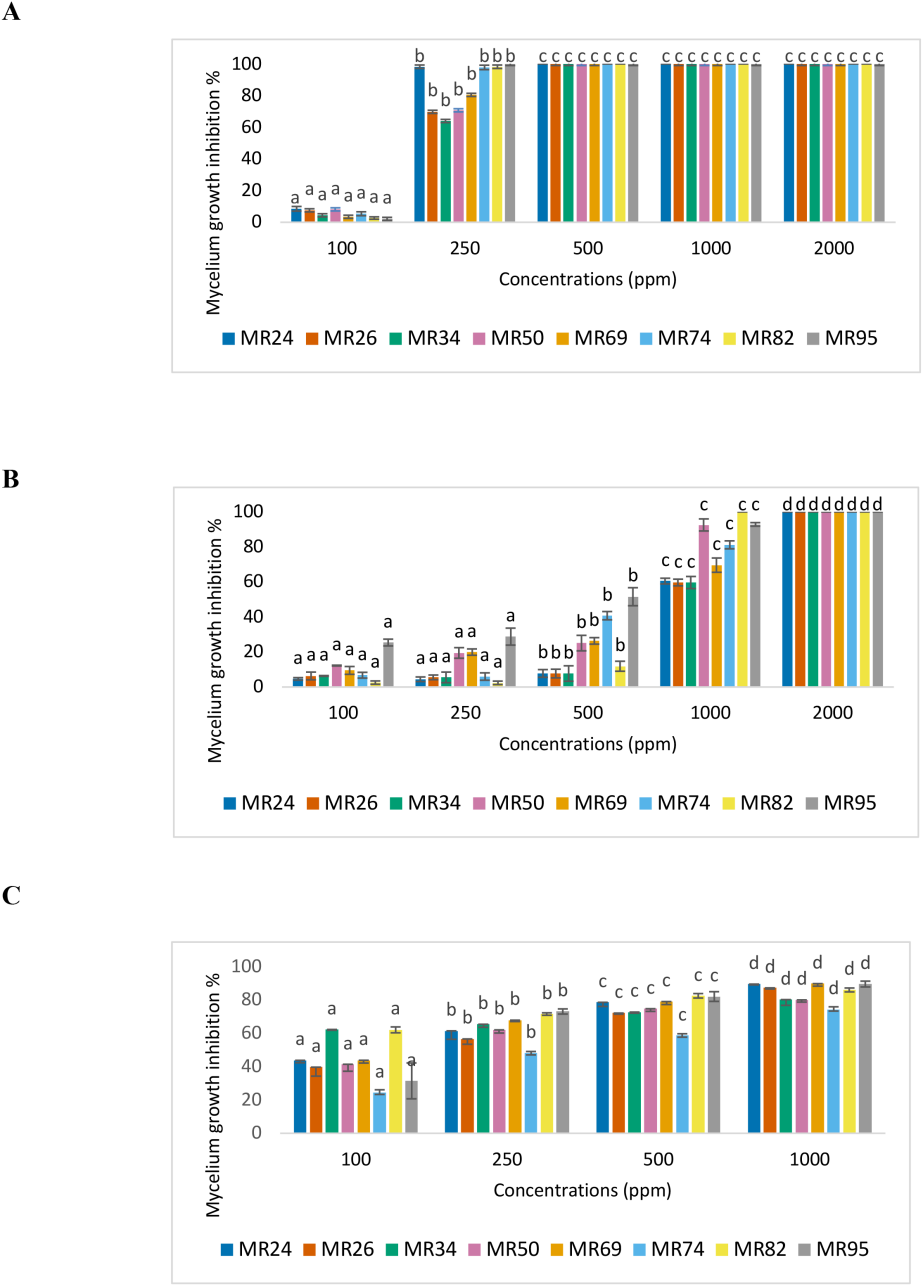
Inhibitory effect on the mycelial growth inhibition of M. roreri. Cinnamon essential oil **(A)**. Peppermint essential oil **(B)**. Fengycin **(C)**. Different letters above the bars indicate statistically significant differences between treatments according to Tukey’s HSD test (p< 0.05).

**Figure 3A** shows the inhibitory effect of cinnamon EO on the mycelial growth of eight *M. roreri* strains (MR24, MR26, MR34, MR50, MR69, MR74, MR82, and MR95), revealing a clear concentration and strain-dependent response (see **Supplementary Figure S1** in the supplementary material).

At 100 ppm, all strains exhibited low inhibition rates (below 20%), with no statistically significant differences between them. This suggests that cinnamon oil exerts a minimal antifungal effect across all strains at low doses. At 250 ppm, a greater differentiation between strains was observed. Strains MR26, MR34, MR50, and MR69 showed significantly higher sensitivity (inhibition ranging from ~70% to 95%), while other strains, such as MR24 and MR95, already reached inhibition levels close to 100%. This variation suggests that certain strains possess lower resistance thresholds, potentially due to differences in membrane composition or enzymatic detoxification capacity.

At 500 ppm and above (1000 and 2000 ppm), all strains were completely inhibited (100%), indicating a fungicidal effect regardless of strain. The consistency of inhibition from 500 ppm onward suggests that this is the minimum effective concentration for total suppression across a genetically diverse population of *M. roreri.* The saturation of the antifungal effect at these concentrations may be attributed to a threshold beyond which cinnamaldehyde, the major active compound, disrupts fungal membranes and metabolic pathways irreversibly.

These strain-specific responses at intermediate concentrations (especially 250 ppm) are particularly relevant when designing integrated control strategies. The variability in sensitivity justifies the later combination of cinnamon oil with other antifungal agents, such as fengycin or peppermint oil, to broaden the spectrum of action and reduce the risk of resistance.

Furthermore, these results underscore the importance of considering intra-species diversity in fungal pathogens when evaluating natural product-based control strategies. Not all *M. roreri* strains respond equally to a given antifungal, which supports the rationale for formulating combinations that provide additive or synergistic effects, thereby enhancing efficacy and durability of the treatment.

These differences highlight the variability in antifungal susceptibility among *M. roreri* strains and the complexity of the resistance mechanisms involved. The observed tolerance in some strains, particularly at intermediate concentrations, suggests the possible presence of adaptive responses, such as alterations in membrane permeability, active efflux of toxic compounds, or enzymatic degradation of bioactive molecules. These intrinsic or acquired resistance traits pose a significant challenge for disease management, mainly when relying on a single mode of action.

Therefore, understanding strain level responses is crucial for designing targeted and sustainable antifungal strategies. This includes the selection of compounds with complementary mechanisms of action and the combination of natural antifungals, such as EOs and biosurfactants, to enhance efficacy and reduce the likelihood of resistance development. Ultimately, the variability among *M. roreri* strains underscores the necessity of integrated approaches that consider pathogen diversity as a key factor in formulating of biocontrol solutions.

**Figure 3B** shows the antifungal activity of peppermint EO at concentrations ranging from 100 to 2000 ppm. The results demonstrate a progressive and significant dose-dependent inhibitory effect (p< 0.05), although the magnitude of inhibition varied considerably among strains (see **Supplementary Figure S2** in the supplementary material).

At 100 ppm, peppermint oil exhibited limited antifungal activity, with inhibition percentages below 20% for all strains. The strain MR95 showed the highest inhibition (~21%), while MR26, MR34, MR50, and MR69 exhibited particularly low susceptibility, suggesting a baseline tolerance to peppermint oil at low concentrations.

When the concentration was increased to 250 ppm, a slight improvement in inhibition was observed. However, the overall effect remained moderate, especially in strains MR26, MR34, MR50, and MR69, which again showed resistance patterns similar to those observed at 100 ppm. This may be associated with their ability to tolerate or neutralize components such as menthol or menthone at sublethal levels. At 500 ppm, the oil produced a more pronounced inhibitory effect, especially on strains MR24, MR82, and MR95, which surpassed 40% inhibition. However, MR50 and MR34 continued to display limited growth suppression, reinforcing the notion of strain-specific resistance mechanisms.

A marked antifungal effect was evident at 1000 ppm, where all strains showed inhibition levels between 60% and 85%. The strains MR24, MR82, and MR95 responded particularly well, indicating higher sensitivity to increased doses. At this concentration, the responses of MR34 and MR50 remained significantly lower than those of the other strains. At the maximum concentration (2000 ppm), peppermint oil achieved complete inhibition (100%) of mycelial growth in all strains, confirming its broad-spectrum efficacy when applied at high concentrations.

**Figure 3C** presents the inhibitory effect of fengycin on the mycelial growth of eight *M. roreri* strains, showing a concentration-dependent antifungal activity (see **Supplementary Figure S3** in the supplementary material). The effectiveness of fengycin increased with higher concentrations, reaching a maximum inhibition of 84.57% at 1000 ppm. No data were available for 2000 ppm due to experimental constraints. At the lowest concentration (100 ppm), inhibition ranged from 30% to 50% across strains, with MR95 showing the highest susceptibility and MR50 and MR74 the lowest. This trend remained consistent at higher concentrations, where MR95 exhibited a notable increase in inhibition, achieving over 80% at 1000 ppm, in contrast to MR34, MR50, and MR74, which showed moderate inhibition values, remaining below 70%. At 250 ppm, most strains showed increased inhibition, particularly MR95 (73.07%) and MR69 (67.86%), suggesting early susceptibility. By 500 ppm, the average inhibition exceeded 70% in five of the eight strains, with MR26, MR69, MR82, and MR95 stood out for their higher sensitivity.

These results highlight that fengycin exhibits broad antifungal activity against *M. roreri*, although the degree of inhibition varies significantly among strains, indicating differential susceptibility. In particular, MR95 consistently showed the highest inhibition values at all concentrations, suggesting it is the most susceptible strain to fengycin. On the contrary, MR34, MR50, and MR74 demonstrated greater tolerance, requiring higher concentrations to achieve comparable inhibitory effects, and thus can be considered more resistant.

### 3.4 Antifungal activity of emulsions (fengycin + EOs)

In the search for effective biological solutions and considering that the tested strains exhibit different sensitivities to the evaluated compounds at concentrations ranging from 100 to 1000 ppm, the proposed strategy was to combine these products based on the formulations described in **Table 2**. Therefore, the evaluated concentrations were 100 and 250 ppm for the F-C mixtures. Meanwhile, a concentration of 1000 ppm was tested for the F-P mixture.

**Table 2:** Proportional combinations (C1-C5) of fengycin and EOs used in mixture-based synergy assays.

Combination	C1	C2	C3	C4	C5
Fengycin 1000 ppm/Cinnamon 100 ppm	0/100	25/75	50/50	75/25	100/0
Fengycin 1000 ppm/Cinnamon 250 ppm	0/100	25/75	50/50	75/25	100/0
Fengycin 1000 ppm/Peppermint 1000 ppm	0/100	25/75	50/50	75/25	100/0

Each combination represents a fixed-ratio mixture, where the two components were mixed in the following proportions (fengycin%/EO%): C1 = 0/100, C2 = 25/75, C3 = 50/50, C4 = 75/25, and C5 = 100/0. These combinations were used to evaluate potential synergistic, additive, or antagonistic interactions between fengycin and the different EOs.

These combinations were designed to enhance antifungal efficacy by leveraging the distinct inhibitory effects observed for each compound individually. These mixtures were formulated to explore potential synergistic effects that could improve overall fungal inhibition and address the variability in strain resistance. In this context, to assess the role of synergism in the antimicrobial activity of the formulations, the FIC was calculated using the Equation 2.

**Figure 4:**
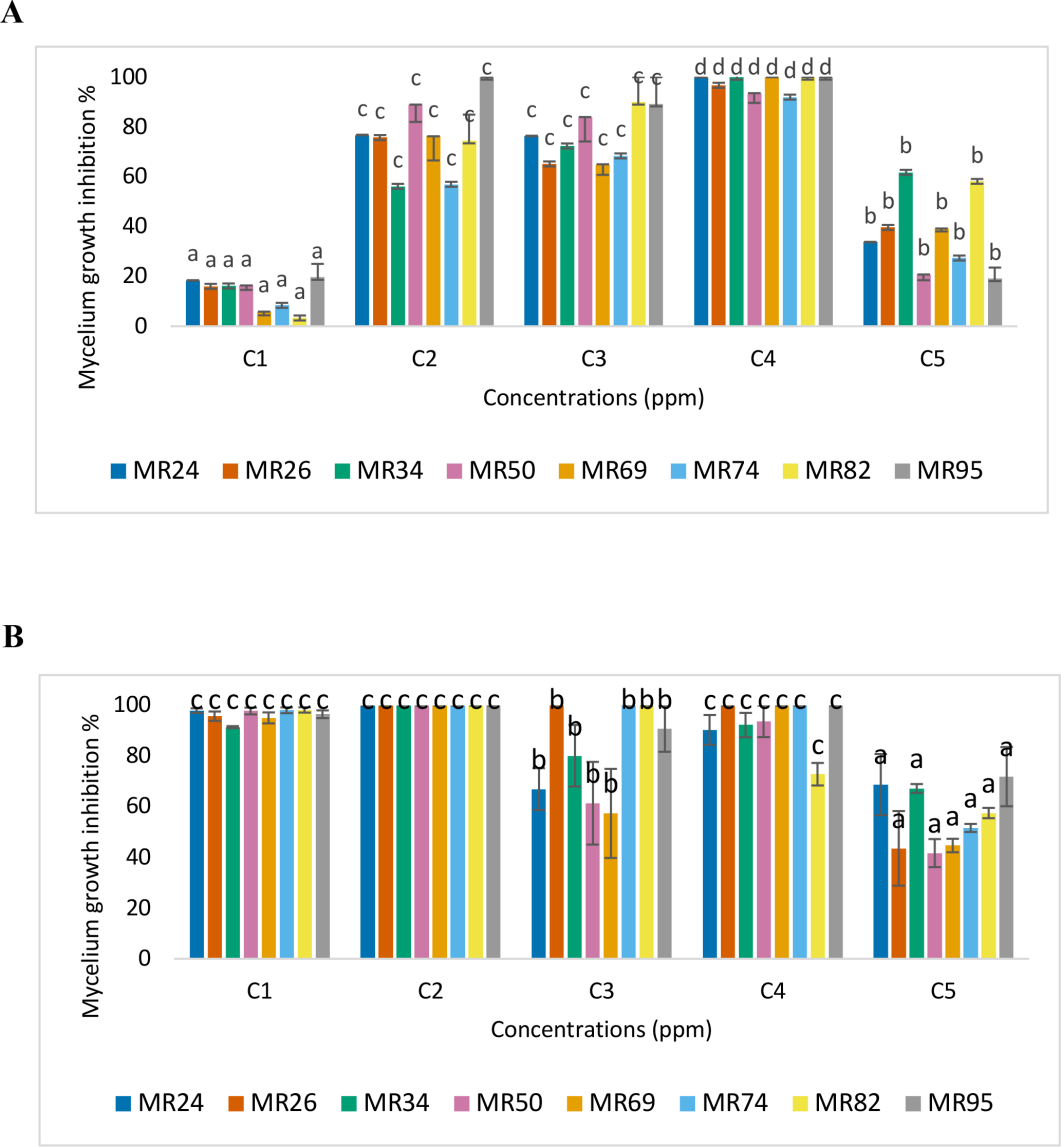
Antifungal activity of mixtures of cinnamon essential oil and fengycin at 100 ppm **(A)** and 250 ppm **(B)** against the mycelial growth of M. roreri. Bars represent mean inhibition percentages across eight fungal strains. Columns labeled with the same letter are not significantly different according to Tukey’s HSD test (p< 0.05).

The effects of the emulsions of F-C at 100 and 250 ppm concentrations showed different inhibitory effects depending on the combinations tested (**Figures 4A, B**). A synergistic effect is evident depending on the strain tested and the concentration used (**Supplementary Table S3**). For instance, at a concentration of 100 ppm (**Figure 4A**), the C4 mixture achieved over 90% inhibition across all strains. Calculation of the FIC value confirmed synergy (FIC< 0.5), indicating that the presence of both compounds enhances antifungal activity compared to their individual effects. However, it is important to note that complete inhibition was not achieved for strains MR26 MR50, and MR74 in the C4 mixture, suggesting that these microorganisms exhibit some resistance under specific conditions.

On the other hand, when using a reference concentration of 250 ppm (**Figure 4B**), the C2 mixture achieved 100% inhibition across all tested strains. In this case, the C2 mixture acted synergistically in controlling the growth of strains MR26, MR34, MR50, and MR69. However, strains MR24, MR74, MR82, and MR95 were found to be more sensitive to the effect of cinnamon alone. FIC calculations indicated no synergy was observed with fengycin (FIC > 0.5) for these strains. These findings highlight the importance of selecting the appropriate mixture and concentration based on the target strain, and the potential for cinnamon to exert significant antifungal effects independently.

**Figure 5:**
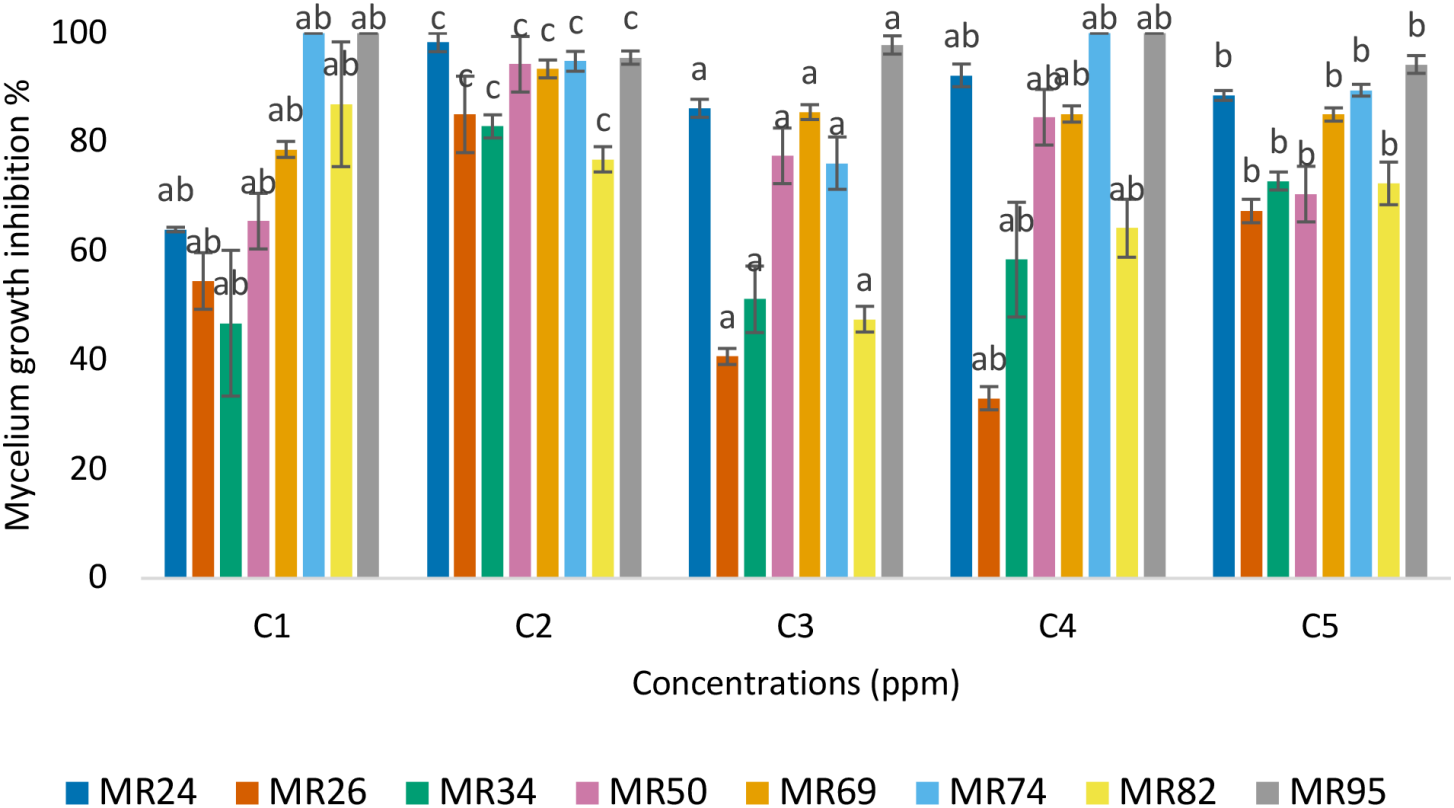
Antifungal activity of mixtures of peppermint essential oil and fengycin at concentration 1000 ppm on mycelial growth of M. roreri. Mean values with the same letter are not significantly different according to Tukey’s HSD test (p< 0.05).

In the case of F-P mixtures, the action varies depending on the combination and *M. roreri* strain. As shown in **Figure 5**, different combinations (C1 to C5) affected mycelial growth inhibition to varying degrees. For instance, mixture C2 provokes inhibition above 90% in strains MR24, MR50, MR69, MR74, and MR95.

The antifungal activity observed across the different treatments reveals that certain *M. roreri* strains exhibit resistance when exposed to individual compounds, particularly at lower concentrations. For instance, strains such as MR26 and MR34 consistently showed partial inhibition and failed to reach complete (100%) suppression when treated with either cinnamon EO, peppermint EO, or fengycin alone. These findings suggest an inherent resistance or tolerance to the individual bioactive components. However, applying formulations, specifically the emulsified combinations of fengycin and cinnamon or peppermint EO, enhanced antifungal performance, even against strains previously identified as less sensitive. For example, in the C2 mixture (F-C at 250 ppm), strains like MR50 and MR69, which required higher concentrations of individual compounds for effective control, were completely inhibited. Similarly, strain MR95, which exhibited high sensitivity to fengycin alone, also showed improved inhibition in the combined treatment, reinforcing the consistent efficacy of the mixture.

Most notably, the mixture C2 induced inhibition rates above 90% in strains such as MR24, MR50, MR69, MR74, and MR95, many of which did not achieve such high levels of inhibition with independent treatments. This outcome suggests that combining fengycin with EOs can overcome certain resistance mechanisms, possibly through complementary action modes targeting different cellular pathways or structures in the fungal cells. Such synergistic or additive effects, even in the absence of formal synergy (as indicated by an FIC of 1.0), contribute to enhanced overall effectiveness. Furthermore, this approach offers practical advantages. Mixtures can achieve desired inhibitory effects at lower concentrations of each component, potentially reducing the risk of toxicity, environmental impact, and development of resistance. It also allows for broader-spectrum control across diverse *M. roreri* strains, accommodating strain-specific variability in sensitivity.

### 3.5 Characterization of antimicrobial emulsions and their stability

The combination of antimicrobial agents is a key strategy in combating antimicrobial resistance. This study, observed significant biological activity in F-C mixtures across all tested *M. roreri* strains. In contrast, F-P mixtures required higher concentrations to achieve antimicrobial effects, and no synergistic interaction was observed between their components.

**Figure 6:**
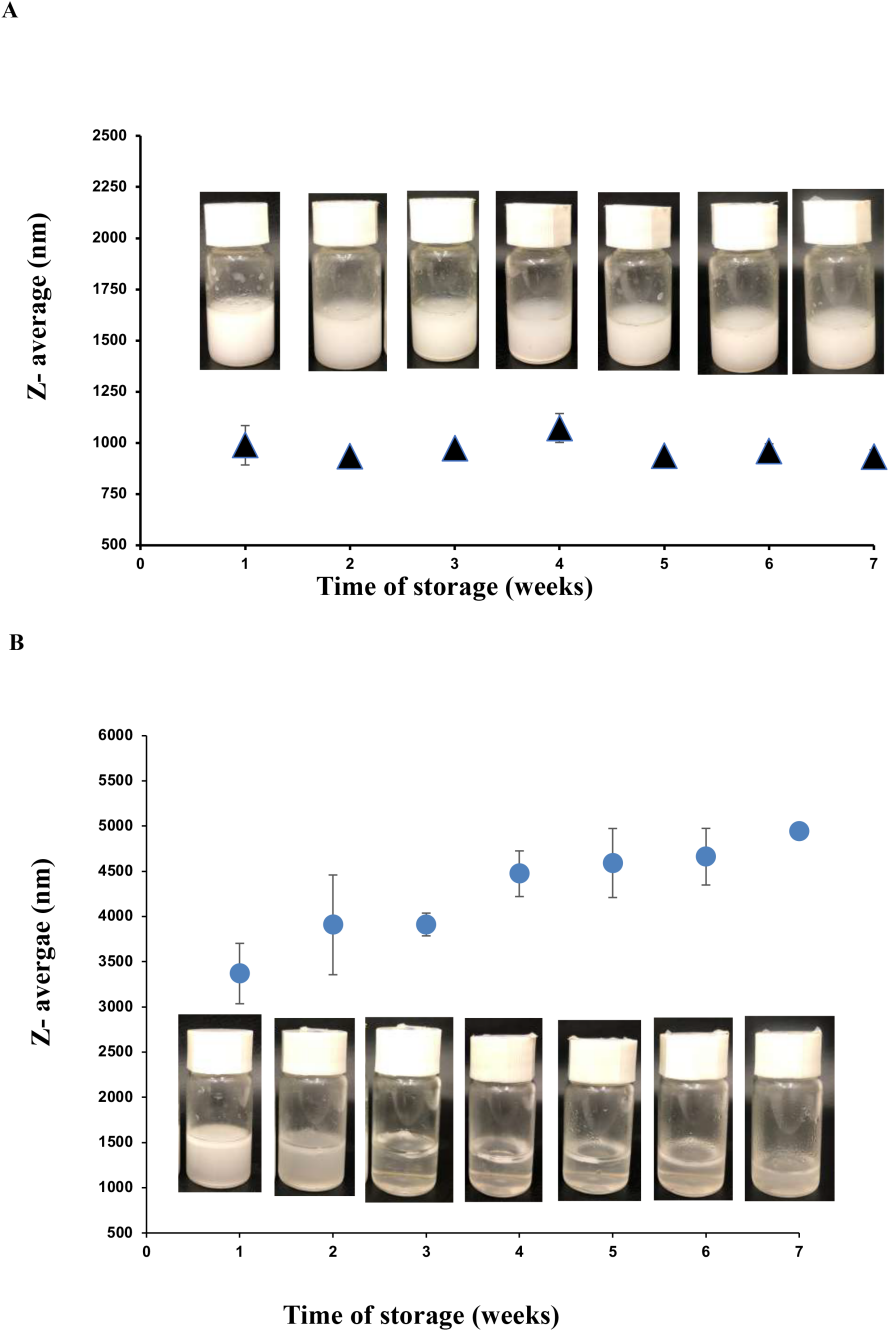
Physicochemical characterization and stability of antimicrobial emulsions combining fengycin with cinnamon (F–C) or peppermint (F–P) essential oils. Evolution of particle size (Z-average) over a 7-week storage period. The F–C emulsion maintained consistent size (850–1050 nm), indicating good colloidal stability **(A)**, while the F–P emulsion showed progressive particle aggregation exceeding 5000 nm by week 7 **(B)**. Zeta potential measurements across six weeks, reflecting electrostatic stability. The F–C emulsion-maintained values between –26.43 mV and –35.67 mV, supporting high stability. In contrast, F–P emulsions exhibited greater fluctuation and a drop to –22.5 mV in week 6, suggesting reduced stability and increased aggregation risk.

To better understand the physicochemical properties that may underlie these differences, we analyzed the particle size (Z-average), zeta potential, and temporal stability of both F-C and F-P emulsions. **Figure 6A** shows the evolution of particle size in these emulsions over a 7-week storage period. The F-C emulsion maintained a relatively consistent particle size, fluctuating between 850 and 1050 nm, indicating good colloidal stability. Conversely, the F-P emulsion exhibited continuous particle growth, exceeding 5000 nm by the seventh week, indicative of progressive aggregation (**Figure 6B**).

These contrasting behaviors likely influence the antimicrobial performance of the formulations. The stability observed in F-C emulsions suggests a more uniform and sustained dispersion of bioactive compounds, enhancing their bioavailability and interaction with fungal cells, potentially explaining the strong antifungal activity and observed synergy. In contrast, the rapid aggregation seen in F-P formulations may limit the availability of active ingredients, necessitating higher concentrations to achieve inhibition and precluding synergistic effects.

Zeta potential analysis further supports these findings. This parameter, derived from electrophoretic mobility, reflects the surface charge of colloidal particles and is critical to predicting emulsion stability. Values exceeding ±20 mV are generally indicative of electrostatic stability, as similarly charged particles repel each other, preventing aggregation (Coronel-León et al., 2017). For F-C emulsions, zeta potential values ranged between -26.43 mV and -35.67 mV over six weeks, suggesting a stable, negatively charged colloidal system with minimal variation. This correlates well with the stable particle size and reinforces the notion of sustained dispersion and functional activity. In contrast, F-P emulsions fluctuated more, with zeta potentials ranging from -22.5 mV to -36.68 mV. Notably, a marked drop to -22.5 mV in week six, and higher standard deviations, indicate diminishing electrostatic stability consistent with the observed particle aggregation trend.

Together, these results emphasize the critical role of physicochemical stability in the antimicrobial efficacy of emulsions. The superior stability of F-C emulsions likely enhances the distribution and sustained interaction of fengycin and cinnamon compounds with fungal cells, contributing to their potency and synergistic effects. Conversely, the reduced stability of F-P formulations may lead to decreased bioavailability and limited efficacy.

In addition to effects on vegetative fungal growth, evaluating antifungal activity against spores is crucial, as spores are often more resistant and play a key role in fungal persistence and dissemination. We therefore extended our analysis to assess the inhibition of spore germination.

The tested emulsions exhibited strong antifungal activity against *M. roreri* spores. Notably, the F-C and F-P emulsions (formulation C2) achieved complete inhibition (100%) of spore germination, suggesting a fungicidal mode of action. In contrast, fengycin alone (formulation C5) showed a concentration-dependent effect: 84.05% inhibition at 100 ppm, increasing to 93.73% at 250 ppm, and 97.07% at 1000 ppm. These results indicate a fungistatic effect at lower doses and fungicidal activity at higher concentrations.

The emulsions’ stability, confirmed through *in vitro* evaluations every 24 hours, supports their long-term antifungal potential. Across all time points, consistent inhibition levels were observed, particularly in formulations combining fengycin with EOs. This sustained activity reinforces the synergistic benefits of such combinations. Thus, the results highlight the advantages of combining fengycin with EOs to enhance antifungal efficacy. While fengycin alone possesses notable bioactivity, its performance significantly improves when combined with cinnamon or peppermint EOs. This synergy results in higher inhibition rates, prolonged activity, and more reliable protection against fungal pathogens. Given their demonstrated stability and effectiveness, these bioactive emulsions represent a promising, sustainable biocontrol strategy for managing fungal infections in crops like cacao, offering an eco-friendly alternative to synthetic fungicides.

### 3.6 Comparative efficacy of fengycin–EOs emulsions on spore germination inhibition

**Figure 7:**
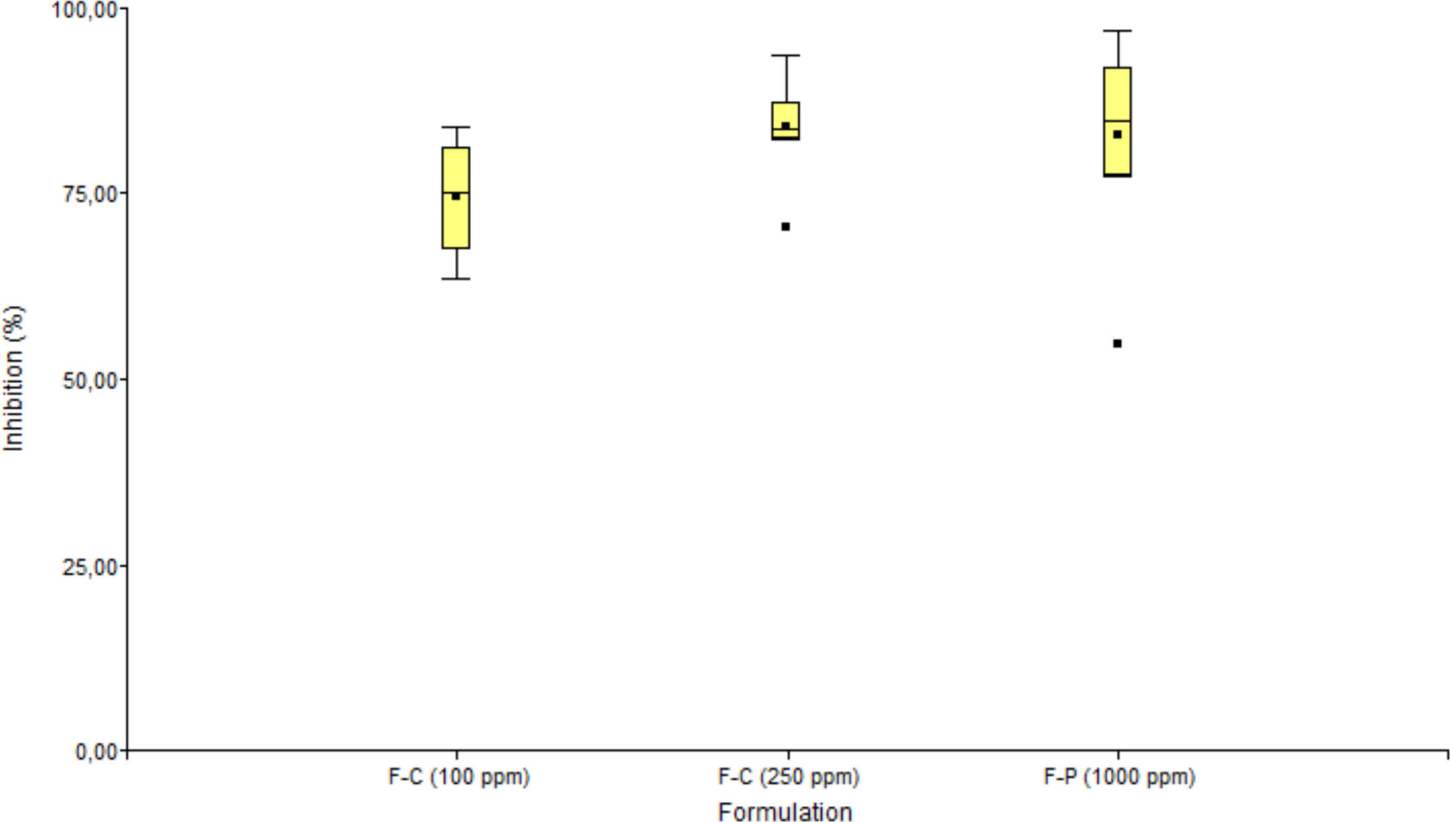
Boxplot showing the inhibition of spore germination of *M. roreri* strains treated with different combinations of fengycin and essential oils (cinnamon and peppermint) at various concentrations. The boxes represent the interquartile range (IQR), the horizontal line within each box indicates the median, and the dots represent outliers. The analysis highlights differences in antifungal effectiveness among formulations, with F-C (250 ppm) showing the highest median inhibition and lowest variability.

The effect of different formulations combining fengycin with EOs on the inhibition of *M. roreri* spore germination was evaluated at three distinct concentration levels: 100 ppm (F–C 100), 250 ppm (F–C 250), and 1000 ppm (F–P 1000). Five treatments (C1–C5), representing various ratios of fengycin and EO, were tested against eight genetically diverse *M. roreri* strains. The results are summarized in **Figure 7**. Representative images of spore germination under each treatment condition are provided in **Supplementary Material Figure S4**.

In formulations F-C at 100 and 250 ppm, treatments C1 to C4 resulted in complete inhibition (100%) of spore germination across all tested strains. These combinations contained both fengycin and EO in varying ratios, highlighting the robust antifungal performance of the synergistic mixtures. In contrast, treatment C5 corresponding to fengycin alone exhibited significantly lower inhibitory activity, with marked differences depending on the formulation and strain.

The boxplot illustrates the spore germination inhibition (%) achieved by three formulations: F–C (100 ppm), F–C (250 ppm), and F–P (1000 ppm). The F–C formulation at 250 ppm and F–P at 1000 ppm exhibited the highest median inhibition values (above 80%), whereas F–C at 100 ppm showed a slightly lower median of around 75%. Moderate variability was observed within each treatment group, and a few outliers were detected, particularly in F–C (250 ppm) and F–P (1000 ppm). These results suggest that the formulation’s type and concentration significantly influence antifungal efficacy. Statistical analysis (ANOVA followed by Tukey’s test, p< 0.05) confirmed significant differences between treatments.

These results underscore the importance of EOs, particularly cinnamon, in enhancing the antifungal efficacy of fengycin. The performance of fengycin alone (C5) was consistently inferior, indicating that synergistic or additive interactions with EO components are critical for successfully suppressing spore germination. The superior performance of the F–C 250 and F–P 1000 formulations further suggests that concentration and type of EO play essential roles in determining treatment efficacy.

## 4 Discussion

The result of this study demonstrates the antifungal potential of fengycin and cinnamon EO against *M. roreri*, both individually and in combinations. Fengycin inhibited mycelial growth up to 84.6% at 1000 ppm, although its efficacy varied among strains (e.g., MR95 was highly susceptible, while MR34 and MR50 showed lower sensitivity). Cinnamon EO exhibited the highest antifungal activity, achieving more than 80% inhibition at 250 ppm and complete inhibition at concentrations ≥500 ppm. Its strong effect is largely attributed to its major compound, (E)-cinnamaldehyde (69.72%), which disrupts membrane integrity, interferes with enzyme activity, and inhibits spore germination. Eugenol (4.02%) also contributes through protein denaturation and increased membrane permeability (Castro Pereira et al., 2022; Taheri et al., 2023).

In contrast, peppermint EO required substantially higher concentrations to reach comparable inhibition levels. Its activity is linked to oxygenated monoterpenes such as menthol, menthone, eucalyptol, and menthyl acetate, which affect membrane structure and metabolic processes. However, the lower efficacy at sublethal doses and the strong strain-dependent variability suggest the presence of adaptive mechanisms in certain *M. roreri* isolates, including detoxification systems, altered membrane permeability, efflux activity, or modified cellular targets (Hu et al., 2021).

Synergistic interactions were observed in F–C emulsions at 100–250 ppm (FIC< 0.5), producing near-complete inhibition in several strains. Nevertheless, some isolates exhibited partial tolerance, likely linked to geographic origin or historical exposure to antifungal compounds (Ruiz-Chután et al., 2024). In contrast, F–P emulsions showed mainly additive effects (FIC ≈ 1), limiting their effectiveness at lower concentrations. Notably, strains MR26 and MR34 consistently displayed reduced sensitivity to cinnamon EO, peppermint EO, and fengycin, indicating a broad tolerance phenotype that may involve membrane remodeling, enhanced detoxification, or restricted compound penetration.

Mass spectrometry confirmed the presence of fengycin isoforms (1435–1491 Da), consistent with reported variants A and B (Farzand et al., 2019; Hentati et al., 2019; Su et al., 2020; Zhu et al., 2023) and clearly distinct from surfactin (1007–1035 Da) or iturin (~1080 Da) (Mardanova et al., 2017). This supports the role of fengycin in the antifungal activity observed, although contributions from other metabolites cannot be excluded entirely. Its mechanism of integrating into sterol-rich membranes and altering permeability has been described previously (Pedraza et al., 2020).

Beyond direct antimicrobial activity, fengycin acted as a biosurfactant, reducing surface tension and stabilizing emulsions of essential oils. This dual role enhances the dispersion of hydrophobic molecules such as cinnamon oil and contributes to the superior performance of F–C formulations. Similar effects of *Bacillus* lipopeptides improving the solubility and bioavailability of bioactive compounds have been reported (Zhou et al., 2016; Guillén-Navarro et al., 2023). Thus, *Bacillus*-derived lipopeptides are considered key elements in bioproduct development (Rodríguez-Chávez et al., 2019), their effectiveness depends strongly on the extraction and formulation methods (Valenzuela-Ruíz et al., 2020).

Physicochemical analyses confirmed that F–C emulsions preserved droplet size and a stable negative zeta potential (≈ –20 mV) for seven weeks, whereas peppermint emulsions aggregated and lost stability (≈ –17 mV). These parameters directly influenced bioactivity, highlighting the central role of formulation stability (Xie et al., 2021).

The complete inhibition of spore germination achieved with the combined formulations indicates a fungicidal mode of action acting at early developmental stages of *M. roreri*, highlighting the effectiveness of combining fengycin with cinnamon EO to control both vegetative and reproductive structures. The differential responses among isolates from distinct Ecuadorian regions reveal marked strain-dependent variability, likely associated with genetic or phenotypic resistance mechanisms such as changes in membrane composition, cell wall structure, or detoxification capacity. These results support the use of combined antifungal strategies to broaden the control spectrum and overcome reduced susceptibility in tolerant strains.

In summary, fengycin characterization is essential to distinguish its contribution from other *Bacillus* lipopeptides and to optimize its application in formulations. Combining fengycin and cinnamon EO offers a sustainable alternative to synthetic fungicides for cacao disease management. In addition, broader applications in pharmaceuticals, surface science, and food preservation can be explored (Ziani et al., 2011; Haba et al., 2014; Coronel-León et al., 2017). In the agricultural sector, future work should prioritize *vivo* assays, field trials, and molecular characterization of *M. roreri* populations to refine and adapt formulations to diverse agroecosystems.

## 5 Conclusion

This study demonstrates that emulsions combining fengycin with cinnamon and peppermint essential oils (EOs) represent a promising strategy for the biological control of *Moniliophthora roreri*, the causal agent of frosty pod rot in cacao. Among the formulations, F–C emulsions were the most effective, inhibiting both mycelial growth and spore germination, and showing synergistic effects in selected strain–concentration combinations (FIC ≤ 0.5). In contrast, F–P emulsions were less stable and exhibited mainly additive effects.

The mixture ratio and the fungal strain evaluated strongly influenced the antifungal performance. Cinnamon EO, combined with fengycin, consistently enhanced inhibition at lower concentrations, whereas strain-specific variability related to the geographic origin of the isolates suggests the importance of considering pathogen diversity when designing biocontrol formulations.

Furthermore, the physicochemical stability of F–C emulsions over seven weeks reinforces their practical applicability. The confirmation of fengycin through mass spectrometry and its dual role as antifungal and biosurfactant underscores its potential as a cornerstone molecule in sustainable formulations.

Overall, fengycin–EO emulsions offer an effective and eco-friendly alternative to chemical fungicides for frosty pod rot management. Future research should focus on greenhouse and field trials to validate long-term efficacy, crop safety, and scalability under real-world cacao production conditions.

## Data Availability

The raw data supporting the conclusions of this article will be made available by the authors, without undue reservation.

## References

[B1] Amaya-Márquez D. J. Espinoza-Lozano R. F. del Castillo D. S. Villavicencio-Vásquez M. E. Pérez-Martínez S. (2021). Inhibition and stimulation of mycelial growth of *Moniliophthora roreri* by flutolanil in populations of Ecuador. Acta Agronomica 70, 240–248. doi: 10.15446/acag.v70n3.88905

[B2] Bailey B. A. Evans H. C. Phillips-Mora W. Ali S. S. Meinhardt L. W. (2018). *Moniliophthora roreri*, causal agent of cacao frosty pod rot. Mol. Plant Pathol. 19, 1580–1594. doi: 10.1111/mpp.12648, PMID: 29194910 PMC6638017

[B3] Bidaud A. L. Schwarz P. Herbreteau G. Dannaoui E. (2021). Techniques for the assessment of *in vitro* and *in vivo* antifungal combinations. J. Fungi 7, 1–16. doi: 10.3390/jof7020113, PMID: 33557026 PMC7913650

[B4] Castro Pereira F. Silva A. de Souza E. Ferreira de Oliveira D. Rodrigues Macedo W. Humberto Silva G. (2022). Cinnamomum cassia essential oil and (E)-cinnamaldehyde as control agents of anthracnose on common bean seeds. J. Phytopathol. 170, 414–421. doi: 10.1111/jph.13092

[B5] Chamba K. P. Pardo D. M. Cabrera L. G. León L. A. (2024). Ecuador: Producción agrícola de cacao de la Economía Popular y Solidaria con respecto al rendimiento por hectárea 2002-2022. Arandu UTIC 11, 1668–1680. doi: 10.69639/arandu.v11i2.274

[B6] Chang Y. Harmon P. F. Treadwell D. D. Carrillo D. Sarkhosh A. Brecht J. K. (2022). Biocontrol potential of essential oils in organic horticulture systems: from farm to fork. Front. Nutr. 8. doi: 10.3389/fnut.2021.805138, PMID: 35096947 PMC8792766

[B7] Chen Y. Li B. Zhang Z. Tian S. (2017). Pathogenicity assay of penicillium expansum on apple fruits. Bio-protocol 7, 635. doi: 10.21769/BioProtoc.2264, PMID: 34541250 PMC8410388

[B8] Coronel-León J. de Grau G. Grau-Campistany A. Farfan M. Rabanal F. Manresa A. . (2015). Biosurfactant production by AL 1.1, a *Bacillus licheniformis* strain isolated from Antarctica: production, chemical characterization and properties. Ann. Microbiol. 65, 2065–2078. doi: 10.1007/s13213-015-1045-x

[B9] Coronel-León J. Pinazo A. Pérez L. Espuny M. J. Marqués A. M. Manresa A. (2017). Lichenysin-geminal amino acid-based surfactants: Synergistic action of an unconventional antimicrobial mixture. Colloids Surfaces B: Biointerfaces 149, 38–47. doi: 10.1016/j.colsurfb.2016.10.008, PMID: 27718395

[B10] Dahunsi S. O. Osueke C. O. Olayanju T. M. A. Lawal A. I. (2019). Co-digestion of *Theobroma cacao* (Cocoa) pod husk and poultry manure for energy generation: Effects of pretreatment methods. Bioresource Technol. 283, 229–241. doi: 10.1016/j.biortech.2019.03.093, PMID: 30913431

[B11] Espinoza-Lozano F. Amaya-Márquez D. Pinto C. M. Villavicencio-Vásquez M. Sosa Del Castillo D. Pérez-Martínez S. (2022). Multiple introductions of *moniliophthora roreri* from the amazon to the pacific region in Ecuador and shared high azoxystrobin sensitivity. Agronomy 12, 1–13. doi: 10.3390/agronomy12051119

[B12] Ezziyyani M. Pérez Sánchez C. Requena E. Sid A. María A. Candela E. . (2004). Evaluación del biocontrol de Phytophthora capsici en pimiento (*Capsicum annuum* L.) por tratamiento con *Burkholderia cepacia*. Anales Biología 26, 61–68.

[B13] Farzand A. Moosa A. Zubair M. Khan A. R. Massawe V. C. Tahir H. A. S. . (2019). Suppression of sclerotinia sclerotiorum by the induction of systemic resistance and regulation of antioxidant pathways in tomato using fengycin produced by *Bacillus amyloliquefaciens* FZB42. Biomolecules 9, 1–17. doi: 10.3390/biom9100613, PMID: 31623124 PMC6843208

[B14] Guato-Molina J. J. Auhing-Arcos J. A. Crespo-Ávila J. A. Esmeraldas-García G. A. Mendoza-León A. F. Canchignia-Martínez H. F. (2019). Plant growth promoting bacteria with potential biocontrol agent of *Fusarium oxysporum* f. Sp. Lycopersici, andMoniliophthora roreri. Scientia Agropecuaria 10, 393–402. doi: 10.17268/sci.agropecu.2019.03.10

[B15] Guillamon E. Martín B. D. Mut-Salud N. Ochando-Pulido J. M. Morales-González J. A. Arjona A. B. . (2024). Optimization of an onion oil microemulsion by response surface methodology for enhanced physicochemical stability and biological activity. LWT 194, 1–10. doi: 10.1016/j.lwt.2024.115809

[B16] Guillén-Navarro K. López-Gutiérrez T. García-Fajardo V. Gómez-Cornelio S. Zarza E. de la Rosa-García S. . (2023). Broad-Spectrum Antifungal, Biosurfactants and Bioemulsifier Activity of *Bacillus subtilis* subsp. *spizizenii*—A Potential Biocontrol and Bioremediation Agent in Agriculture. Plants 12, 1–20. doi: 10.3390/plants12061374, PMID: 36987062 PMC10056679

[B17] Haba E. Bouhdid S. Torrego-Solana N. Marqués A. M. Espuny M. J. García-Celma M. J. . (2014). Rhamnolipids as emulsifying agents for essential oil formulations: Antimicrobial effect against *Candida albicans* and methicillin-resistant *Staphylococcus aureus*. Int. J. Pharmaceutics 476, 134–141. doi: 10.1016/j.ijpharm.2014.09.039, PMID: 25269010

[B18] Hentati D. Chebbi A. Hadrich F. Frikha I. Rabanal F. Sayadi S. . (2019). Production, characterization and biotechnological potential of lipopeptide biosurfactants from a novel marine *Bacillus stratosphericus* strain FLU5. Ecotoxicology Environ. Saf. 167, 441–449. doi: 10.1016/j.ecoenv.2018.10.036, PMID: 30384057

[B19] Hu M. Chen S. Schnabel G. Walker A.-S. (2021). Non-target site mechanisms of fungicide resistance in crop pathogens: A review. Microorganisms 9, 502. doi: 10.3390/microorganisms9030502, PMID: 33673517 PMC7997439

[B20] Jiménez D. L. Alvarez J. C. Mosquera S. (2022). Frosty pod rot: a major threat to cacao plantations on the move. Trop. Plant Pathol. 47, pp. doi: 10.1007/s40858-021-00472-y

[B21] Kongor J. E. Owusu M. Oduro-Yeboah C. (2024). Cocoa production in the 2020s: challenges and solutions. CABI Agric. Bioscience 5, 1–28. doi: 10.1186/s43170-024-00310-6

[B22] Lam-Gutiérrez A. Ayora-Talavera T. Garrido-Ramírez E. R. Ruíz-Valdiviezo V. M. Guzmán-Albores J. M. Cristóbal-Alejo J. (2024). Chemical composition and antifungal activity of essential oils extracted from Pimenta dioica and Piper auritum leaves grown in Mexico. Cogent Food Agric. 10, 1–11. doi: 10.1080/23311932.2024.2356935

[B23] Mardanova A. M. Fanisovna G. Tafkilevich M. Valerevna I. Farvazovna L. Gadelevna A. . (2017). *Bacillus subtilis* Strains with Antifungal Activity against the Phytopathogenic Fungi. Agric. Sci. 08, pp. doi: 10.4236/as.2017.81001

[B24] Martins G. A. Bicas J. L. (2024). Antifungal activity of essential oils of tea tree, oregano, thyme, and cinnamon, and their components. Braz. J. Food Technol. 27, 1–5. doi: 10.1590/1981-6723.07123

[B25] Mendoza I. C. Ortiz E. Dreher M. Villavicencio M. Coello D. Chuchuca G. . (2022). Conventional and non-conventional disinfection methods to prevent microbial contamination in minimally processed fruits and vegetables. LWT 165, 1–19. doi: 10.1016/j.lwt.2022.113714, PMID: 35783661 PMC9239846

[B26] Mohammed A. Mohammad A. Javed A. (2022). Investigation of factors influencing formation of nanoemulsion by spontaneous emulsification: impact on droplet size, polydispersity index, and stability. Bioengineering 9, 1–17. doi: 10.3390/bioengineering9080384, PMID: 36004909 PMC9404776

[B27] Pedraza L. A. López C. E. Uribe-Vélez D. (2020). Mechanisms of action of Bacillus spp. (Bacillaceae) against phytopathogenic microorganisms during their interaction with plants. Acta Biologica Colombiana 25, 112–125. doi: 10.15446/abc.v25n1.75045

[B28] Porcino M. M. Oliveira V. de S. da Silva H. F. de Souza M. D. S. Do Nascimento L. C. (2023). Essential oils in the management of Alternaria alternata f. sp. *citri* in ‘Dancy’ tangerine fruits. Rev. Caatinga 36, 291–299. doi: 10.1590/1983-21252023v36n206rc

[B29] Ribes S. Fuentes A. Talens P. Barat J. M. (2017). Application of cinnamon bark emulsions to protect strawberry jam from fungi. LWT 78, 265–272. doi: 10.1016/j.lwt.2016.12.047

[B30] Rodríguez-Chávez J. L. Juárez-Campusano Y. S. Delgado G. Pacheco Aguilar J. R. (2019). Identification of lipopeptides from *Bacillus* strain Q11 with ability to inhibit the germination of *Penicillium expansum*, the etiological agent of postharvest blue mold disease. Postharvest Biol. Technol. 155, 72–79. doi: 10.1016/j.postharvbio.2019.05.011

[B31] Ruiz-Chután J. A. Berdúo-Sandoval J. E. Alvarado V. Kalousová M. Lojka B. Ziarovská J. . (2024). Genetic diversity and population structure of *Moniliophthora roreri* in cocoa producing areas of Guatemala. J. microbiology Biotechnol. Food Sci. 20, e5947. doi: 10.55251/jmbfs.5947

[B32] Sanchez-Tamayo M. Plaza-Dorado J. L. Ochoa-Martínez C. (2024). Influence of composite edible coating of pectin, glycerol, and oregano essential oil on postharvest deterioration of mango fruit. Food Sci. Nutr. 12, 10646–10654. doi: 10.1002/fsn3.4545, PMID: 39723088 PMC11666998

[B33] Shaaban H. (2020). “ Essential oil as antimicrobial agents: efficacy, stability, and safety issues for food application,” in Essential oils - bioactive compounds, new perspectives and applications (London, United Kingdom: IntechOpen). doi: 10.5772/intechopen.92305

[B34] Silva M. da G. C. Medeiros A. O. Converti A. Almeida F. C. G. Sarubbo L. A. (2024). Biosurfactants: promising biomolecules for agricultural applications. Sustainability (Switzerland) 16, 1–32. doi: 10.3390/su16010449

[B35] Su Z. Chen X. Liu X. Guo Q. Li S. Lu X. . (2020). Genome mining and UHPLC–QTOF–MS/MS to identify the potential antimicrobial compounds and determine the specificity of biosynthetic gene clusters in *Bacillus subtilis* NCD-2. BMC Genomics 21, 1–16. doi: 10.1186/s12864-020-07160-2, PMID: 33153447 PMC7643408

[B36] Taheri P. Soweizy M. Tarighi S. (2023). Application of essential oils to control some important fungi and bacteria pathogenic on cereals. J. Nat. Pestic. Res. 6, 100052. doi: 10.1016/J.NAPERE.2023.100052

[B37] Valenzuela-Ruíz V. Villa-Rodriguez E. Parra-Cota F. I. Santoyo G. (2020). Lipopeptides produced by biological control agents of the genus *Bacillus*: a review of analytical tools used for their study. Revista Mexicana Ciencias Agrícolas. 11, 1–15. doi: 10.29312/remexca.v11i2.2191

[B38] Villavicencio-Vásquez M. Espinoza-Lozano F. Espinoza-Lozano L. Coronel-León J. (2025). Biological control agents: mechanisms of action, selection, formulation and challenges in agriculture. Front. Agron. 7. doi: 10.3389/fagro.2025.1578915

[B39] Villegas-Rascón R. E. López-Meneses A. K. Plascencia-Jatomea M. Cota-Arriola O. Moreno-Ibarra G. M. Castillón-Campaña L. G. . (2018). Control of mycotoxigenic fungi with microcapsules of essential oils encapsulated in chitosan. Food Sci. Technol. (Brazil) 38, 335–340. doi: 10.1590/1678-457X.04817

[B40] Xie H. Ni F. Liu C. Shi J. Ren G. Wu Z. . (2021). Characterization and stability of peppermint oil emulsions using polyglycerol esters of fatty acids and milk proteins as emulsifiers. J. Food Sci. 86, 5148–5158. doi: 10.1111/1750-3841.15952, PMID: 34755898

[B41] Yánez-Mendizábal V. Falconí C. E. (2021). *Bacillus subtilis* CtpxS2–1 induces systemic resistance against anthracnose in Andean lupin by lipopeptide production. Biotechnol. Lett. 43, 719–728. doi: 10.1007/s10529-020-03066-x, PMID: 33389271

[B42] Yin Y. Miao J. Shao W. Liu X. Zhao Y. Ma Z. (2023). Fungicide resistance: progress in understanding mechanism, monitoring, and management. Phytopathology 113, 707–718. doi: 10.1094/PHYTO-10-22-0370-KD, PMID: 36624725

[B43] Yu J. (2025). Chemical composition of essential oils and their potential applications in postharvest storage of cereal grains. Molecules 30. doi: 10.3390/molecules30030683, PMID: 39942787 PMC11820458

[B44] Zhou H. Luo C. Fang X. Xiang Y. Wang X. Zhang R. . (2016). Loss of GltB inhibits biofilm formation and biocontrol efficiency of *Bacillus subtilis* Bs916 by altering the production of ã-polyglutamate and three lipopeptides. PloS One 11, 1–20. doi: 10.1371/journal.pone.0156247, PMID: 27223617 PMC4880196

[B45] Zhu H. Wu S. Tang S. Xu J. He Y. Ren Z. . (2023). Isolation, identification and characterization of biopotential cyclic lipopeptides from *Bacillus subtilis* strain JN005 and its antifungal activity against rice pathogen *Magnaporthe oryzae*. Biol. Control 182, 105241. doi: 10.1016/j.biocontrol.2023.105241

[B46] Ziani K. Chang Y. McLandsborough L. McClements D. J. (2011). Influence of surfactant charge on antimicrobial efficacy of surfactant-stabilized thyme oil nanoemulsions. J. Agric. Food Chem. 59, 6247–6255. doi: 10.1021/jf200450m, PMID: 21520914

