# Supplementary material for: Fengycin–essential oil emulsions as sustainable biocontrol formulations against Moniliophthora roreri, the cacao frosty pod rot pathogen
Source: Front Plant Sci. 2026 Jan 16;16:1731535. doi: 10.3389/fpls.2025.1731535 (PMC12855478; doi:10.3389/fpls.2025.1731535)
Supplement: Supplementary file 1 [file Table1.docx]

**Supplementary Table S1. Chemical composition of cinnamon essential oil**

| **Class** | **Compounds** | **Peak area (%) ± SD ^a^** | **Retention index (estimated) ^b^** | **Retention index (reference) ^c^** |  |
| --- | --- | --- | --- | --- | --- |
| Styrene | Cinnamene | 0.02 ± 0.00 | 888.92 | 891.00 |  |
| Monoterpene | α-Thujene | 0.12 ± 0.00 | 925.66 | 928.00 |  |
| Monoterpene | α-Pinene | 0.62 ± 0.00 | 932.13 | 933.00 |  |
| Monoterpene | Camphene | 0.25± 0.00 | 946.42 | 943.00 |  |
| Aldehyde | Benzaldehyde | 0.30 ± 0.00 | 956.30 | 959.00 |  |
| Monoterpene | β-Pinene | 0.24 ± 0.00 | 974.77 | 970.00 |  |
| Monoterpene | α-Phellandrene | 0.76 ± 0.00 | 1003.36 | 1010.00 |  |
| Monoterpene | δ-3-Carene | 0.08 ± 0.00 | 1009.21 | 1011.00 |  |
| Monoterpene | α-Terpinene | 0.37 0.00 | 1015.45 | 1017.00 |  |
| Monoterpene | o-Cymene | 1.86 ± 0.00 | 1023.24 | 1025.40 |  |
| Monoterpene | β-Phellandrene | 2.41 ± 0.00 | 1027.80 | 1030.00 |  |
| Monoterpene | β-cis-Ocimene | 0.02 ± 0.00 | 1036.96 | 1039.00 |  |
| Monoterpene | γ-Terpinene | 0.04 ± 0.00 | 1057.55 | 1059.00 |  |
| Ketone | Acetophenone | 0.02 ± 0.00 | 1063.12 | 1072.00 |  |
| Monoterpene | α-Terpinolene | 0.12 ± 0.00 | 1087.29 | 1089.00 |  |
| Oxygenated monoterpene | β-Linalool | 3.27 ± 0.01 | 1099.38 | 1098.00 |  |
| Oxygenated monoterpene | D-Camphor | 0.27 ± 0.00 | 1141.88 | 1141.00 |  |
| Aldehyde | Benzenepropanal | 0.20 ± 0.00 | 1159.54 | 1160.00 |  |
| Oxygenated monoterpene | L-Borneol | 0.08 ± 0.00 | 1163.79 | 1166.00 |  |
| Oxygenated monoterpene | 4-Terpineol | 0.28 ± 0.00 | 1175.61 | 1176.00 |  |
| Oxygenated monoterpene | α-Terpineol | 0.46 ± 0.00 | 1188.75 | 1189.00 |  |
| Aldehyde | Cinnamaldehyde | 0.22 ± 0.00 | 1216.39 | 1232.00 |  |
| Aldehyde | (E)-Cinnamaldehyde | 69.72 ± 0.15 | 1280.99 | 1268.00 |  |
| Benzodioxole | Safrole | 0.38 ± 0.00 | 1289.56 | 1289.00 |  |
| Oxygenated monoterpene | Carvacrol | 0.07 ± 0.00 | 1300.90 | 1298.00 |  |
| Alcohol | Cinnamic alcohol | 0.19 ± 0.01 | 1303.92 | 1304.00 |  |
| Phenylpropene | Eugenol | 4.02 ± 0.02 | 1358.28 | 1363.00 |  |
| Sesquiterpene | α-Copaene | 0.69 ± 0.01 | 1376.67 | 1376.00 |  |
| Sesquiterpene | β-Elemene | 0.04 ± 0.00 | 1392.43 | 1398.00 |  |
| Sesquiterpene | β-Caryophyllene | 5.05 ± 0.02 | 1423.95 | 1424.00 |  |
| Ester | Cinnamyl acetate | 2.72 ± 0.03 | 1450.13 | 1441.00 |  |
| Sesquiterpene | α-Humulene | 0.93 ± 0.00 | 1461.34 | 1456.00 |  |
| Sesquiterpene | α-Curcumene | 0.04 ± 0.00 | 1493.43 | 1481.00 |  |
| Sesquiterpene | Ledene | 0.03 ± 0.00 | 1506.29 | 1499.00 |  |
| Sesquiterpene | α-Muurolene | 0.02 ± 0.00 | 1510.87 | 1502.00 |  |
| Sesquiterpene | δ-Cadinene | 0.09 ± 0.00 | 1531.75 | 1537.00 |  |
| Aldehyde | Cinnamaldehyde, o-methoxy- | 0.27 ± 0.01 | 1534.53 | 1512.00 |  |
| Oxygenated sesquiterpene | Caryophyllene oxide | 0.67 ± 0.00 | 1584.49 | 1575.00 |  |
| Aldehyde | Myristaldehyde | 0.37 ± 0.00 | 1610.89 | 1601.00 |  |
| Ester | Benzyl benzoate | 0.76 ± 0.00 | 1761.07 | 1765.00 |  |
| Alcohols |  | 0.19 |  |  |  |
| Aldehydes |  | 71.08 |  |  |  |
| Benzodioxoles |  | 0.38 |  |  |  |
| Esters |  | 3.47 |  |  |  |
| Ketones |  | 0.02 |  |  |  |
| Monoterpenes |  | 6.90 |  |  |  |
| Oxygenated monoterpenes |  | 4.43 |  |  |  |
| Sesquiterpenes |  | 6.89 |  |  |  |
| Oxygenated sesquiterpenes |  | 0.67 |  |  |  |
| Phenylpropenes |  | 4.02 |  |  |  |
| Styrenes |  | 0.02 |  |  |  |
| Total |  | 98.09 |  |  |  |
| ^a^ Mean value (n=3) ± standard deviation (SD) | |  |  |  | |
| ^b^ Estimated values in capillary column HP-5 | |  |  |  | |
| ^c^ Reference values estimated in non-polar capillary column. | |  |  |  | |

**Supplementary Table S2. Chemical composition of peppermint essential oil**

| **Class** | **Compounds** | **Peak area (%) ± SD ^a^** | | **Retention index (estimated) ^b^** | **Retention index (reference) ^c^** |
| --- | --- | --- | --- | --- | --- |
| Ketone | 3-Methylcyclopentanone | 0.01 ± 0.00 | | 842.11 | 832.00 |
| Monoterpene | α-Pinene | 0.36 ± 0.00 | | 932.27 | 933.00 |
| Monoterpene | Camphene | 0.03 ± 0.00 | | 946.66 | 943.00 |
| Monoterpene | Sabinene | 0.16 ± 0.00 | | 972.03 | 964.00 |
| Monoterpene | β-Pinene | 0.43 ± 0.00 | | 975.01 | 970.00 |
| Monoterpene | β-Myrcene | 0.06 ± 0.00 | | 990.40 | 981.00 |
| Alcohol | 3-Octanol | 0.09 ± 0.00 | | 994.29 | 985.00 |
| Monoterpene | δ-3-Carene | 0.02 ± 0.00 | | 1009.42 | 1011.00 |
| Monoterpene | o-Cymene | 0.61 ± 0.00 | | 1023.28 | 1025.40 |
| Oxygenated monoterpene | Eucalyptol | 6.06 ± 0.01 | | 1030.05 | 1032.00 |
| Oxygenated monoterpene | trans-Sabinene hydrate | 0.05 ± 0.00 | | 1065.38 | 1060.00 |
| Oxygenated monoterpene | Isopulegol | 1.05 ±0.01 | | 1144.79 | 1146.00 |
| Oxygenated monoterpene | Menthone | 17.12 ± 0.01 | | 1155.24 | 1153.00 |
| Oxygenated monoterpene | Neoisomenthol | 10.58 ± 0.01 | | 1164.98 | 1164.00 |
| Oxygenated monoterpene | Menthol | 51.07 ± 0.01 | | 1179.37 | 1175.00 |
| Oxygenated monoterpene | Isomenthol | 0.84 ± 0.01 | | 1184.68 | 1182.00 |
| Oxygenated monoterpene | α-Terpineol | 0.68 ± 0.00 | | 1191.05 | 1192.00 |
| Oxygenated monoterpene | cis-p-mentha-1(7), 8-dien-2-ol | 0.03 ± 0.00 | | 1218.31 | 1235.00 |
| Oxygenated monoterpene | Pulegone | 2.34 ± 0.01 | | 1238.78 | 1237.00 |
| Oxygenated monoterpene | Carvone | 0.11 ± 0.01 | | 1242.49 | 1243.00 |
| Oxygenated monoterpene | Piperitone | 0.97 ± 0.01 | | 1252.93 | 1253.00 |
| Alcohol | 1-Decanol | 0.10 ± 0.00 | | 1271.61 | 1272.00 |
| Oxygenated monoterpene | Isomenthol acetate | 0.12 ± 0.00 | | 1275.13 | 1282.00 |
| Oxygenated monoterpene | Menthyl acetate | 4.18 ± 0.01 | | 1294.36 | 1294.00 |
| Sesquiterpene | Ylangene | 0.05 ± 0.00 | | 1371.83 | 1370.00 |
| Sesquiterpene | α-Copaene | 0.04 ± 0.00 | | 1376.48 | 1376.00 |
| Sesquiterpene | β-Bourbonene | 0.22 ± 0.00 | | 1385.39 | 1386.00 |
| Sesquiterpene | Isolongifolene | 0.04 ± 0.00 | | 1387.83 | 1387.00 |
| Sesquiterpene | β-Elemene | 0.08 ± 0.00 | | 1392.38 | 1398.00 |
| Sesquiterpene | β-Caryophyllene | 0.25 ± 0.00 | | 1422.30 | 1424.00 |
| Sesquiterpene | β-Cubebene | 0.03 ± 0.00 | | 1433.34 | 1434.00 |
| Sesquiterpene | α-Muurolene | 0.04 ± 0.00 | | 1460.77 | 1440.00 |
| Sesquiterpene | γ-Muurolene | 0.02 ± 0.00 | | 1486.94 | 1471.00 |
| Sesquiterpene | δ-Cadinene | 0.02 ± 0.00 | | 1531.84 | 1537.00 |
| Oxygenated sesquiterpene | Viridiflorol | 0.03 ±0.00 | | 1591.84 | 1594.00 |
| Alcohols |  | 0.19 | |  |  |
| Ketones |  | 0.01 | |  |  |
| Monoterpenes |  | 1.66 | |  |  |
| Oxygenated monoterpenes |  | 95.20 | |  |  |
| Sesquiterpenes |  | 0.79 | |  |  |
| Oxygenated sesquiterpenes |  | 0.03 | |  |  |
| Total |  | 97.88 | |  |  |
| ^a^ Mean value (n=3) ± standard deviation (SD) | |  |  |  |  |
| ^b^ Estimated values in capillary column HP-5 | |  |  |  |  |
| ^c^ Reference values estimated in non-polar capillary column | | |  |  |  |

**Supplementary Table S3. Fractional Inhibitory Concentration (FIC) values of fengycin–EO emulsions against *M. roreri* strains, letters denote interaction type (S = synergistic, NS = no synergism, I = indifferent).**

| **Strain** | **F-C**  **100 ppm** | **F-C**  **250 ppm** | **F-P**  **1000 ppm** |
| --- | --- | --- | --- |
| MR24 | 0,175 (S) | 0,8125 (NS) | 1 (I) |
| MR26 | 0,125 (S) | 0,4375 (S) | 1 (I) |
| MR34 | 0,125 (S) | 0,4375 (S) | 1 (I) |
| MR50 | 0,125 (S) | 0,4375 (S) | 1 (I) |
| MR69 | 0,125 (S) | 0,4375 (S) | 1 (I) |
| MR74 | 0,175 (S) | 0,8125 (NS) | 1 (I) |
| MR82 | 0,175 (S) | 0,8125 (NS) | 1 (I) |
| MR95 | 0,175 (S) | 0,8125 (NS) | 1 (I) |
