# Supplementary material for: Fengycin–essential oil emulsions as sustainable biocontrol formulations against Moniliophthora roreri, the cacao frosty pod rot pathogen
Source: Front Plant Sci. 2026 Jan 16;16:1731535. doi: 10.3389/fpls.2025.1731535 (PMC12855478; doi:10.3389/fpls.2025.1731535)
Supplement: Supplementary file 2 [file DataSheet1.docx]

**Supplementary Figures**


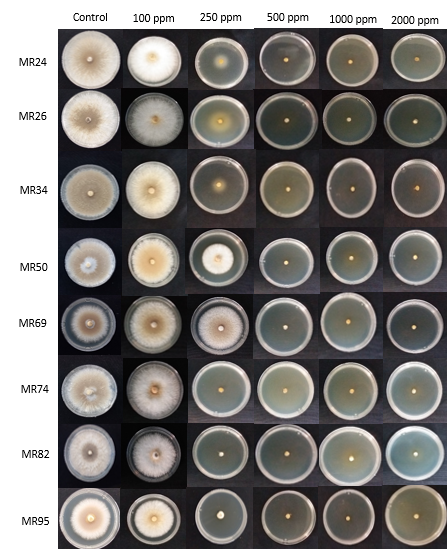


**Figure S1.** M. roreri colonies grown on PDA medium and treated with cinnamon EO at increasing concentrations (100, 250, 500, 1000, and 2000 ppm), after 10 days of incubation at 28 °C. A clear concentration-dependent antifungal effect is observed. Minimal inhibition is evident at 100 ppm, while partial suppression occurs at 250 ppm. Complete inhibition of mycelial growth is achieved at concentrations of 500 ppm and above, confirming the fungicidal activity of cinnamon EO at higher doses.


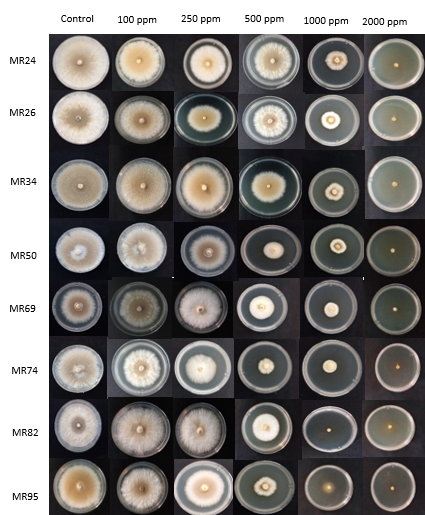


**Figure S2.** M. roreri colonies cultured on PDA medium and treated with peppermint EO at increasing concentrations (100, 250, 500, 1000, and 2000 ppm), after 10 days of incubation at 28 °C. A dose-dependent antifungal response is observed. At lower concentrations (100–250 ppm), fungal growth remains largely unaffected, with only slight reduction in colony diameter. Noticeable inhibition appears at 500 ppm, while near-complete or total suppression of mycelial growth is evident at 1000 and 2000 ppm, indicating strong fungicidal activity at higher doses.


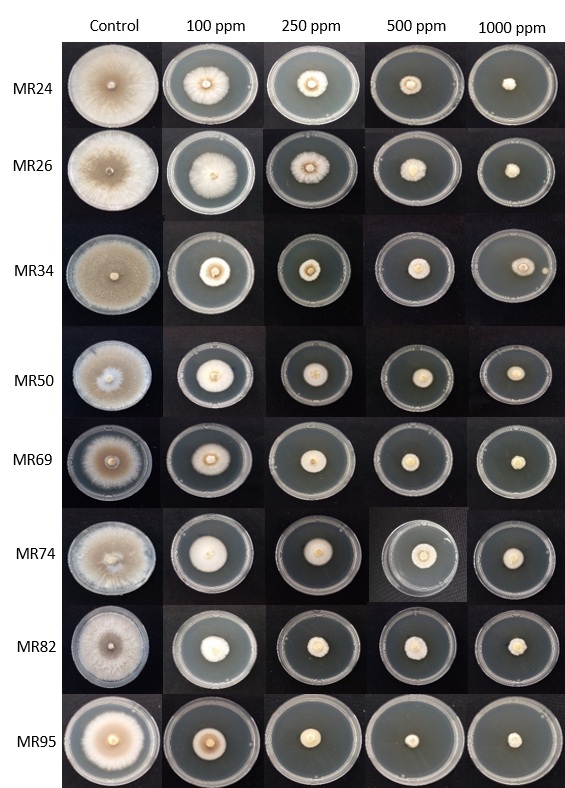


**Figure S3.** M. roreri colonies grown on PDA medium after treatment with fengycin at increasing concentrations (100, 250, 500, and 1000 ppm), incubated at 28 °C for 10 days. Progressive reduction in mycelial growth and colony diameter is observed with increasing fengycin concentration. Partial inhibition is evident at 100 and 250 ppm, while near-complete suppression is achieved at 1000 ppm, indicating a concentration-dependent antifungal effect consistent with quantitative inhibition data presented in the main text.

**A**

**
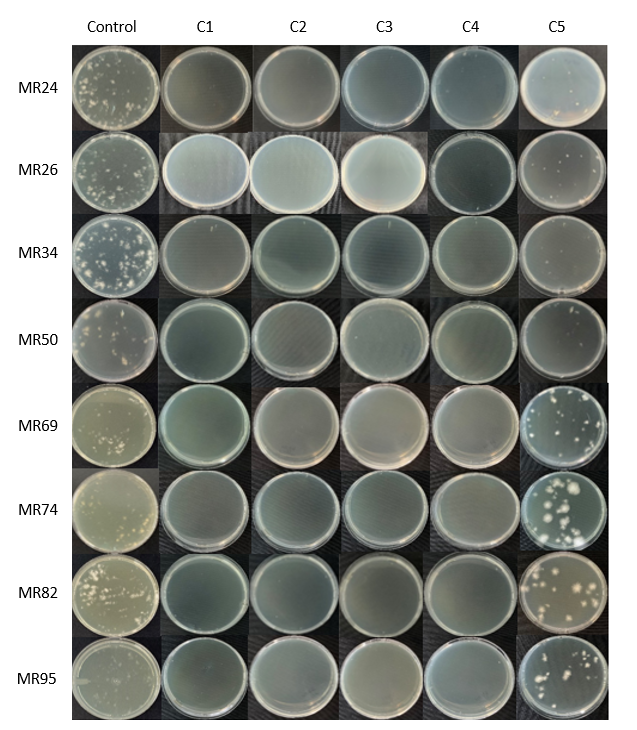
**

**B**


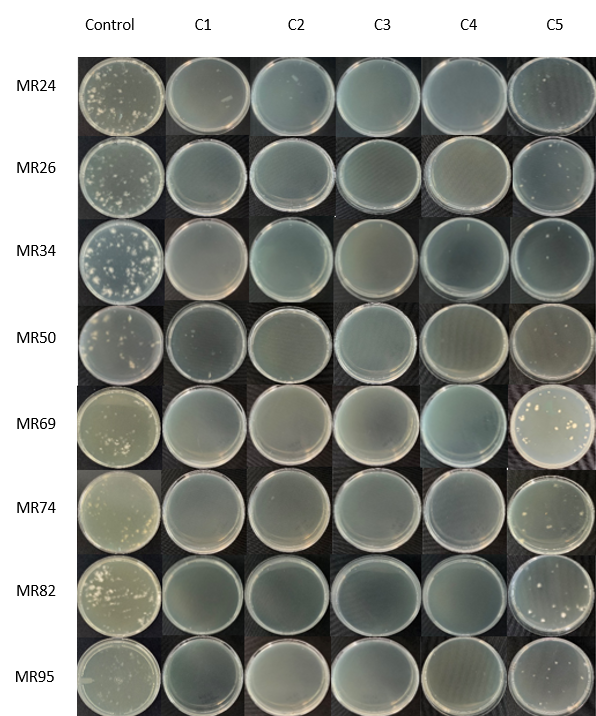


**C**


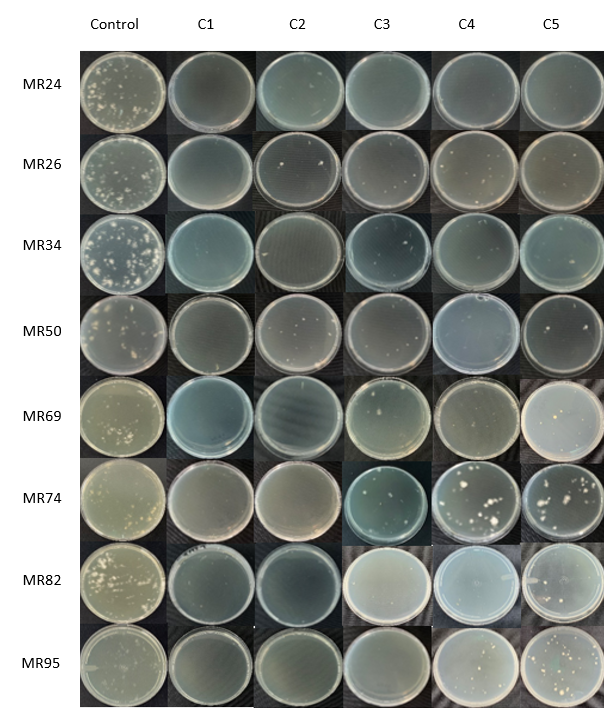


**Figure S4.** Microscopic view of *M. roreri* spores treated with fengycin–essential oil (EO) emulsions after 5 days of incubation at 28 °C on PDA. **(A)** Fengycin (1000 ppm) + cinnamon EO (100 ppm); **(B)** Fengycin (1000 ppm) + cinnamon EO (250 ppm), **(C)** Fengycin (1000 ppm) + peppermint EO (1000 ppm). Across all panels, treatments follow the same proportional combinations: **C1** = 0/100; **C2** = 25/75; **C3** = 50/50; **C4** = 75/25; **C5**=100/0 (fengycin: EO). In all treatments, complete inhibition of spore germination is observed, with no visible germ tubes, indicating a strong fungicidal effect. These images support the quantitative germination inhibition data presented in Figure 7 and confirm the efficacy of combined formulations in suppressing the reproductive structures of *M. roreri*.
